# Distinct effects of global signal regression on brain activity during propofol and sevoflurane anesthesia

**DOI:** 10.3389/fnins.2025.1576535

**Published:** 2025-05-22

**Authors:** Fa Lu, Lunxu Li, Juan Wang, Xuanling Chen, Ho-Ching Yang, Xiaoli Li, Lan Yao, Zhenhu Liang

**Affiliations:** ^1^School of Electrical Engineering, Yanshan University, Qinhuangdao, China; ^2^Key Laboratory of Intelligent Rehabilitation and Neuromodulation of Hebei Province, Yanshan University, Qinhuangdao, China; ^3^Department of Anesthesiology, Peking University International Hospital, Beijing, China; ^4^Department of Radiology and Imaging Sciences, Indiana University School of Medicine, Indianapolis, IN, United States; ^5^State Key Laboratory of Cognitive Neuroscience and Learning and IDG/McGovern Institute for Brain Research, Beijing Normal University, Beijing, China

**Keywords:** fMRI, general anesthesia, global signal, amplitude of low-frequency fluctuation, graph theory

## Abstract

**Introduction:**

Global signal regression (GSR) is widely used in functional magnetic resonance imaging (fMRI) analysis, yet its effects on anesthetic-related brain activity are not well understood.

**Methods:**

Using fMRI data from patients under general anesthesia, we analyzed temporal variability indices, amplitude of low-frequency fluctuations, functional connectivity, and graph theoretical measures with and without GSR.

**Results:**

Here we show that GSR differentially affects brain activity patterns during propofol- and sevoflurane-induced unconsciousness. While temporal variability indices decreased similarly between conscious and unconscious states regardless of GSR, functional connectivity analyses revealed anesthetic-specific effects: GSR altered specific network connections under propofol but broadly reduced connectivity differences under sevoflurane. Network topology analyses demonstrated that GSR minimally affected propofol-induced changes in graph theoretical measures but significantly diminished sevoflurane-related network alterations.

**Discussion:**

These findings reveal that GSR’s impact on functional brain organization is anesthetic-specific, with sevoflurane-induced changes being particularly sensitive to global signal removal. Our results suggest that GSR should be applied cautiously when comparing different anesthetic agents and highlight the importance of considering drug-specific effects when analyzing consciousness-related brain activity.

## 1 Introduction

Exploring the underlying neurophysiological mechanisms of anesthetic-induced loss of consciousness (LOC) remains one of the most challenging questions in the field of neuroscience (Brown et al., 2010). Functional magnetic resonance imaging (fMRI), which provides high spatial resolution for neuroimaging, has been extensively utilized to investigate the mechanisms of consciousness (Liu X. et al., 2017; Huang et al., 2018; Palanca et al., 2015). The blood-oxygen-level-dependent (BOLD) signal derived from fMRI reflects underlying neural activity through neurovascular coupling (Hillman, 2014). However, the precise interpretation of the global signal (GS) in BOLD imaging and its influence on functional connectivity and brain networks during anesthetic-induced unconsciousness remains poorly understood.

The low-pass filtered GS represents the average whole-brain BOLD signal, which reflects low-frequency global fluctuations ranging from 0.001 to 0.1 Hz. Global signal regression (GSR) is a mathematical preprocessing method for fMRI that employs linear regression to remove global effects—including head motion, respiration, and cardiac cycles—from the BOLD signal (Power et al., 2014; Liu T. T. et al., 2017; Murphy and Fox, 2017). However, there have been ongoing debates regarding the implementation and efficacy of GSR in neuroimaging analyses. Some researchers argue that GSR effectively removes global effects and enhances the spatial specificity of connectivity analyses (Fox et al., 2009; Li et al., 2019). Conversely, studies have demonstrated that GSR application to BOLD signals can alter local and long-range correlations (Saad et al., 2012) and may limit the assessment of connectivity patterns (Almgren et al., 2020). Furthermore, recent research has emphasized the critical role of GS in brain–body coupling and its implications for behavioral and cognitive processes (Zhang and Northoff, 2022).

Previous investigations have indicated that loss of consciousness (LOC) induced by anesthesia is attributable to decreased neural activity and diminished information integration among cortical regions (Alkire et al., 2008). Temporal variability quantifies the state-to-state transition capability across temporal scales (i.e., the dynamic shifts in activity patterns across different brain networks; Huang et al., 2016). Additionally, the amplitude of low-frequency fluctuation (ALFF) provides a characterization of regional spontaneous neuronal activity in the frequency domain (Zang et al., 2007; Zuo et al., 2010). These complementary metrics enable the examination of neural activity modifications at both the subject and voxel levels. With retaining GS, Huang et al. empirically demonstrated that the relationship between temporal variability and signal synchronization undergoes significant disruption under general anesthesia (Huang et al., 2016); however, they did not indicate whether this synchronization persists after the GSR. Huang et al. empirically demonstrated, without applying GSR, that the relationship between temporal variability and signal synchronization undergoes significant disruption under general anesthesia. But it remains unknown whether this relationship persists after GSR, highlighting the need to further investigate how GSR affects fMRI indices across different states of consciousness. Considering the controversial nature of GS, a comprehensive systematic analysis is imperative to investigate the influence of GS on temporal variability and ALFF during general anesthesia. Through the analysis we can identify which neural signatures of consciousness states are robust to GSR and which are significantly altered, potentially helping to distinguish more reliable biomarkers of consciousness transitions.

Functional connectivity (FC) is a commonly used method in fMRI that measures the information integration or synchronization between spatially separated brain regions (Friston, 1994). Graph theory-based measures derived from FC have been extensively applied to investigate cognition, consciousness, and mental disorders (Wang et al., 2018; Stanley et al., 2015). In studies of anesthetic-induced unconsciousness, Li et al. demonstrated that both the characteristic path length increased and clustering coefficient decreased from baseline to loss of consciousness (LOC) using electroencephalogram (EEG; Li et al., 2021). Furthermore, both global and local efficiency demonstrated significant decreases from baseline to LOC (Blain-Moraes et al., 2017; Lee et al., 2020). Although network-based analyses have become crucial in consciousness research, few studies have examined the effects of GS on brain networks.

**Figure 1:**
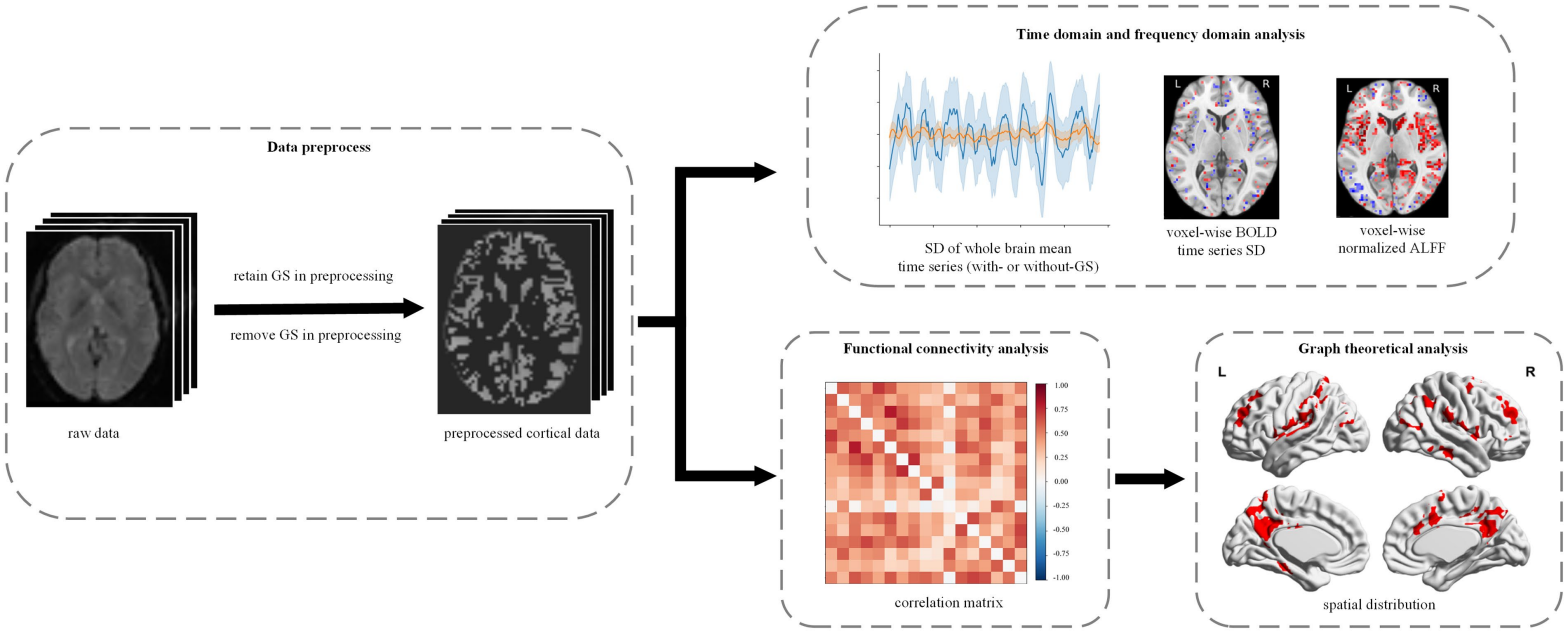
Experimental design and analytical workflow. We studied the effects of GS in propofol- and sevoflurane-induced anesthesia. All the fMRI data were preprocessed with and without GS, respectively. Then, the preprocessed data were used to conduct time domain and frequency domain analysis, FC analysis and graph theoretical analysis.

In this study, we hypothesized that GS plays distinct roles in anesthetic-induced unconscious states and investigated its effect on BOLD signals during general anesthesia. Specifically, we analyzed changes in temporal and frequency domain indices, functional connectivity (FC), and graph theory metrics under anesthesia-induced loss of consciousness (LOC) using two distinct anesthetic agents: propofol (intravenous) and sevoflurane (volatile), both with and without global signal regression (GS; Figure 1). Our findings provide insights into the role of GS in the neural mechanisms underlying consciousness modulation during anesthesia.

## 2 Materials and methods

### 2.1 Participants

This study was approved by Ethics Committee of Peking University International Hospital, Peking University, Beijing, China. Written informed consent was obtained from all participants. The study included a total of 22 glioma patients, with 13 patients receiving propofol anesthesia (male/female: 8/5; mean age ± SD: 42 ± 14.19 years, range: 18 to 67 years) and nine patients with sevoflurane anesthesia (male/female: 3/6; mean age ± SD: 42 ± 43.89 years, range: 20 to 57 years). To reduce the effect of the glioma lesion on the results, nine patients with lesions larger than 30 mm in diameter were excluded. A total of 13 patients, consisting of 7 with propofol anesthesia and 6 with sevoflurane anesthesia were enrolled for further data preprocess and statistical analysis. There were no significant differences in age (*t*-test, *p* = 0.66) or sex distribution (chi-square test, *p* = 0.763) between the two anesthesia groups included in the final analysis.

All patients in this study underwent surgery for supratentorial glioma, which required intraoperative magnetic resonance imaging (MRI)-assisted tumor resection. During the MRI scan, various physiological measurements were continuously monitored, including the electrocardiogram (ECG), heart rate (HR), pulse oxygen saturation (SpO2), non-invasive blood pressure (NBP), end-tidal carbon dioxide (ETCO2) and bispectral index (BIS). To monitor invasive arterial pressure (IBP), ultrasound-guided radial artery puncture was performed. Additionally, two venous accesses were established in the upper extremities for all patients. During induction of general anesthesia, all patients were treated by intravenous injection of propofol (1.5–2 mg/kg), remifentanil (1–2 μg/kg), and rocuronium (0.6–1.0 mg/kg). Oral tracheal intubation was assisted by video laryngoscope, and followed by mechanical ventilation using an anesthesia machine with oxygen flow 2.0 L/min, tidal volume 6–8 mL/kg, respiratory rate 10–16 times/min to maintain blood oxygen saturation 98–100% and ETCO2 35–40 mmHg. Patients were maintained with propofol and remifentanil, or sevoflurane and remifentanil for 30 min, respectively, without additional sedative and analgesic agents (propofol: 1.3–3.5 mg/kg, mean = 2.02, SD = 0.83; sevoflurane: 1 ~ 2.5 MAC, mean = 1.75, SD = 0.52). The electrodes of BIS were withdrawn if the BIS can be maintained at a stable level between 40 and 60 for more than 10 min, and patients were scanned with the same stable effect-site concentration. Intravenous norepinephrine with pump was administered to prevent mean arterial pressure (MAP) from decreasing beyond 20% of the preoperative level during the scan.

### 2.2 fMRI data acquisition

All the anatomical images and functional images were collected through a Siemens 3 T scanner (Siemens Verio Dot 3.0 T, Germany) with an 8-channel phase sensitivity encoding head coil (IMRIS). High-resolution T1-weighted (T1w) anatomical image was acquired for each participant (TR/TE/TI = 2300/3.25/900 ms, FA = 90°, FOV = 250 × 250 mm, image matrix: 256 × 256, 192 slices with 1-mm thickness, gap = 0 mm). Functional images were acquired from whole brain gradient echo-planar images (TR/TE = 220/30 ms, FA = 90°, FOV = 192 × 192 mm, image matrix: 64 × 64). The scan time in both wakefulness and general anesthesia was 540 s.

### 2.3 Data preprocessing

Preprocessing was performed using fMRIPrep 21.0.2 (Esteban et al., 2019) for T1w images mainly including (1) intensity non-uniformity correction for T1w image; (2) brain surface reconstruction; (3) skull-striping and tissue segment; (4) normalization to MNI space. For BOLD images preprocessing mainly includes (1) slice-timing correction; (2) motion correction; (3) resample to the MNI space (detailed in Supplementary material).

After the preprocessing in fMRIPrep, several procedures were implemented through python package Nilearn 0.9.1 (https://nilearn.github.io/) for further preprocessing: (1) the BOLD signals were band-pass filtered between 0.01 Hz and 0.1 Hz and spatial smoothed by using a Gaussian filter of 6 mm FWHM; (2) discarded the first five volumes; (3) motion-related confounds (six parameters) and their derivatives, as well as white matter and cerebrospinal fluid signals, were regressed out of the analysis; (4) time series in gray matter (GM) were extracted and normalized by z-score accounting for the difference in variance of nonneural origin (e.g., distance and head coil). Since our study focused on the GS, the previous confounds regression steps were repeated under two different conditions: with GS and without GS (termed as withGS and withoutGS, respectively).

### 2.4 Temporal variability and ALFF analysis

For each state of each participant, we extracted the average time series across all voxels in GM, also known as the GS. We first calculated the SD of GM average time series. We than calculate voxel-wise SD for every voxel in GM. To obtain ALFF, the voxel-wise BOLD signals in GM were transformed to frequency domain using fast Fourier transform (FFT). The square root of power spectrum was then calculated to obtain the ALFF (Zang et al., 2007). ALFF was then normalized within the range of 0 to 1. Finally, we averaged the voxel-wise SD and normalized ALFF across voxels in GM to obtain the subject-level measures for both voxel-wise SD and normalized ALFF. The same calculation was applied to the datasets with GSR.

### 2.5 Functional connectivity analysis

The Yeo’s 17 functional networks atlas was apply to divide the brain into eight networks: Visual (VisCent, VisPeri), Somatomotor (SomMot), Dorsal Attention (DorsAttn), Salience/Ventral Attention (SalVentAttn), Limbic, Control (Cont), Temporal Parietal (TempPar) and Default mode networks (Yeo et al., 2011). All voxel time series in each network were averaged, resulting in 17 time series corresponding to the different functional networks. To characterize the FC between brain regions, we calculated Pearson’s correlations between these 17 time series. This calculation resulted in a 17 × 17 matrices, referred to as FC matrix. The diagonal elements of the FC matrix, which indicate self-connections, were set to zero. All the subject-level FC matrixes were then averaged into group-level matrixes based on their respective states and anesthetics.

### 2.6 Graph theoretical analysis

Graph theoretical indices were computed using the Brain Connectivity Toolbox^1^ for analyzing the brain network. To characterize brain network, time series in 114 cortical regions of interest (ROIs) that cover Yeo’s 17 functional networks atlas were extracted (Yeo et al., 2011). Referring to previous study (Wang et al., 2018), we applied the cost thresholding method to remove those false connections and ensure that the ROI matrixes are sparse. The cost threshold value is defined as the ratio between the number of edges in the network and all the possible edges, which is based on the criteria below: (1) at most 10% of the nodes are not fully connected in 95% of participants, (2) the average number of connections per node was larger than the log of the number of nodes, and (3) the small worldness of brain networks was > 1. The group-level cost threshold ranges of different states and drugs were then obtained. We used 0.005 as the cost value step size, which allowed us to calculate the graph theoretical indices (Supplementary Table S1). Here we evaluated three nodal level indices (path length, clustering coefficient and local efficiency) and two global level indices (global efficiency and small worldness).

The characteristic path length, which reflects the brain network’s capability for integrating information, is defined as the average shortest path length across all possible pairs of nodes in the network, whereas the shortest path length is defined as the sum of the minimal weights from one node to another:


(1) $$L=\frac{1}{n}\sum_{i\in N}L_{i}=\frac{1}{n}\sum_{i\in N}\frac{\sum_{j\in N,j\neq i}d_{\mathrm{ij}}}{n-1}$$

where *L_i_* is the average distance between node *i* and all other nodes. The *d_ij_* denotes the weighted shortest path length between node *i* and *j*. Then the characteristic path length *L* was normalized:


(2) $$\lambda =\frac{L}{L_{\mathrm{rand}}}$$

Where *λ* represents normalized path length (NPL) and *L_rand_* denotes the weighted characteristic path length of the set of random networks.

Different brain regions can be grouped for specialized information processing, including integration and segregation. Clustering coefficient is a parameter for quantifying the segregation of brain function, which measures the ability of one node interconnect with other nodes. The clustering coefficient of a node is defined as the fraction of triangles around a node and the whole brain’s clustering coefficient is equal to the average clustering coefficient across all nodes:


(3) $$C=\frac{1}{n}\sum_{i\in N}C_{i}=\frac{1}{n}\sum_{i\in N}\frac{2t_{i}}{k_{i}(k_{i}-1)}$$

where *C_i_* is the clustering coefficient of node *i* (*C_i_* = 0 for *k_i_* < 2), *t_i_* denotes the weighted geometric mean of triangles around a node *i*, and *k_i_* represented the degree of node *i*. Then the clustering coefficient *C* was normalized:


(4) $$\gamma =\frac{C}{C_{\mathrm{rand}}}$$

where the *γ* denotes the normalized clustering coefficient (NCC) and *C_rand_* denotes the clustering coefficient of the set of random networks.

Local efficiency is the index for measuring the information transfer of the subgraph induced by the neighbors of the node. Higher local efficiency indicates that the neural information is processed more separately. The local efficiency is defined as the inverse of the shortest average path length of all neighbors of a given node and the whole brain’s local efficiency is the average of all nodes:


(5) $$E_{\mathrm{loc}}=\frac{1}{2}\sum_{i\in N}\frac{\sum_{j,h\in N,j\neq i}{\left(w_{\mathrm{ij}}w_{\mathrm{ih}}{\left[d_{\mathrm{jh}}^{w}(N_{i})\right]}^{-1}\right)}^{1/3}}{k_{i}(k_{i}-1)}$$

where *E_loc_* is the local efficiency of node *i*, *w_ij_* is the weight connection between *i* and *j* and *d_jh_* (*N_i_*) is the length of the shortest path between *j* and *h* which contains only neighbors of *i*.

The global efficiency is the average inverse shortest path length in the network, since paths between disconnected nodes are assumed to have infinite lengths and corresponded to zero efficiency:


(6) $$E_{\text{glob}}=\frac{1}{n}\sum_{i\in N}E_{i}=\frac{1}{n}\sum_{i\in N}\frac{\sum_{j\in N,j\neq i}d_{\mathrm{ij}}^{-1}}{n-1}$$

where *E_i_* is the efficiency of node *i*.

The small world organization of human brain is one of the most important findings of graph theory. The small worldness exhibits the ability of information segregation and integration with low energy and wiring costs. It is defined as the ratio of the normalized clustering coefficient to the normalized path length:


(7) $$\mathrm{SW}=\frac{\gamma }{\lambda }=\frac{C/C_{\mathrm{rand}}}{L/L_{\mathrm{rand}}}$$

A higher small worldness (i.e., a higher clustering coefficient and lower characteristic path length) means the brain network processes information more effectively. A brain network with *SW* > 1 is regarded as having the small-world characteristic. Additionally, to obtain appropriate *L_rand_* and *C_rand_*, for each subject of each state, we generated 100 random networks with the same nodes, edges and degree distribution as the actual network. *L_rand_* and *C_rand_* were evaluated as the averaged characteristic path length and the averaged clustering coefficient of sets of random networks.

### 2.7 Statistical analysis

R project (version 4.2.2; accessed October 31, 2022)^2^ are used for statistical analysis. First, the Bayesian linear mixed model (LMM) in ‘brms’ package (Bürkner, 2017) was applied to analyze the interaction effects of the three factors: (1) state (i.e., baseline and unconscious state), (2) anesthetics (i.e., propofol and sevoflurane), and (3) GS condition (i.e., withGS and withoutGS). Besides, the random intercept for each subject were considered as the random effects. In Bayesian LMM, the median, 95% confidence interval (CI) and probability of direction (pd) were estimated (especially pd. = 97.5% correspond approximately to two-sided *p*-value 0.05; Makowski et al., 2019).

At subject level, we conducted Bayesian paired samples t tests (two-tailed) to compare all the indices between baseline and unconscious state under withGS and withoutGS conditions with default effect size priors and Cauchy scale 0.707. The posterior distribution of Bayesian paired samples t tests was reported with the median and 95% CI. To test the significance, we calculated the two-tailed Bayes factor BF_10_, which quantifying the relative probability of the observed data (i.e., BF_10_ is equal to *p*(data | hypotheses1: there is effect) / *p*(data | hypotheses0: there is no effect)). This approach is particularly advantageous for our study with a limited sample size, as Bayesian methods can effectively detect meaningful differences in small samples while maintaining appropriate uncertainty estimates (Larson et al., 2023). We selected BF₁₀ > 3 as our significance threshold, which indicates that the evidence for the alternative hypothesis is at least 3 times stronger than for the null hypothesis. This threshold was chosen based on two considerations: (1) previous studies showing that BF values for different test statistics fall within the range of 2.4 to 3.4 when p equals 0.05 (Benjamin et al., 2018); and (2) established guidelines suggesting that 3 < BF₁₀ ≤ 10 indicates moderate evidence for the alternative hypothesis (H₁; Etz and Vandekerekhove, 2016).

## 3 Results

### 3.1 Global signal effects on temporal variability and low-frequency fluctuations

We investigated the effects of GS on SD and ALFF during anesthetic-induced general anesthesia. The validation results revealed that there is no significant three-way interaction effect among the factors for SD, voxel-wise SD and ALFF (pd < 97.5%). Subsequently, the three two-way interaction effects were analyzed. Similarly, no significant two-way interaction effects were found in any of the three indicators (pd < 97.5%).

**Figure 2:**
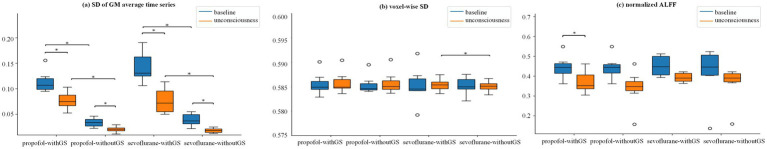
Global signal effects on temporal variability and ALFF measures across consciousness states. **(a–c)** are the Group statistic boxplots of SD GM average time series, voxel-wise SD and normalized ALFF from baseline to unconscious state, respectively, in the condition of withGS and withoutGS. Voxel-wise SD and normalized ALFF are the mean value across the gray-matter (GM). The asterisks refer to significance level (*BF_10_ > 3 between the states) for the Bayesian paired samples t tests (two-tailed). SD, standard deviant; ALFF, amplitude of low-frequency fluctuation; withGS, data processed retaining GS; withoutGS, data processed removing GS.

Figure 2a and Supplementary Tables S2, S3 demonstrates that GS removal led to a significant decrease in the SD of GM average time series in both states for propofol anesthesia (baseline: BF_10_ = 840.12, 0.079 [CI: 0.060, 0.099]; unconscious state: BF_10_ = 192.96, 0.056 [CI: 0.037, 0.076]) and sevoflurane anesthesia (baseline: BF_10_ = 40.79, 0.104 [CI: 0.082, 0.124]; unconscious state: BF_10_ = 32.22, 0.058 [CI: 0.037, 0.079]). Under conditions with GS, there were significant differences in SD of GM average time series between baseline and unconsciousness for both propofol and sevoflurane (BF_10_ = 3.89, 0.037 [CI: 1.64e-06, 0.055] and BF_10_ = 7.41, 0.066 [CI: 4.42e-02, 0.086], respectively). Similarly, the SD of GM average time series without GS also showed significance between baseline and unconscious state for both propofol and sevoflurane (BF_10_ = 7.43, 0.014 [CI: −6.31e-03, 0.033] and BF_10_ = 11.55, 0.021 [CI: 1.07e-06, 0.042], respectively). Figure 2b and Supplementary Tables S4, S5 illustrates the average voxel-wise SD value of all patients, only sevoflurane voxel-wise SD in unconscious state exhibited a significant decrease after removing GS (BF_10_ = 3.10, 3.13e-04 [CI: −0.002, 0.0027]). However, there was no significant difference between baseline and unconscious state for propofol and sevoflurane (BF_10_ < 3). For the average normalized ALFF in Figure 2c, only propofol ALFF without GS significantly decreased after LOC (BF_10_ = 6.35, 0.072 [CI: −0.011, 0.153]; Supplementary Table S6). No significant difference was observed in sevoflurane ALFF although it showed a downward trend in both conditions (withGS and withoutGS; BF_10_ < 3; Supplementary Table S7). Further, we examined the spatial changes of voxel-wise ALFF and SD. In the withGS condition, both anesthetics showed significant decreases in normalized ALFF, particularly in the cingulate gyrus, bilateral precentral gyrus, insular and hippocampus regions, while minimal changes were observed in the withoutGS condition. After false discovery rate correction, neither propofol nor sevoflurane showed any significant regional differences in either voxel-wise ALFF or voxel SD (Supplementary Figures S1–S3).

In contrast to normalized ALFF and voxel-wise SD, the SD of GM average time series demonstrated capability in differentiating between states (baseline and unconscious state), while the remaining indices (normalized ALFF and voxel-wise SD) exhibited limited statistical significance.

### 3.2 Anesthetic-specific alterations in functional connectivity following global signal regression

**Figure 3:**
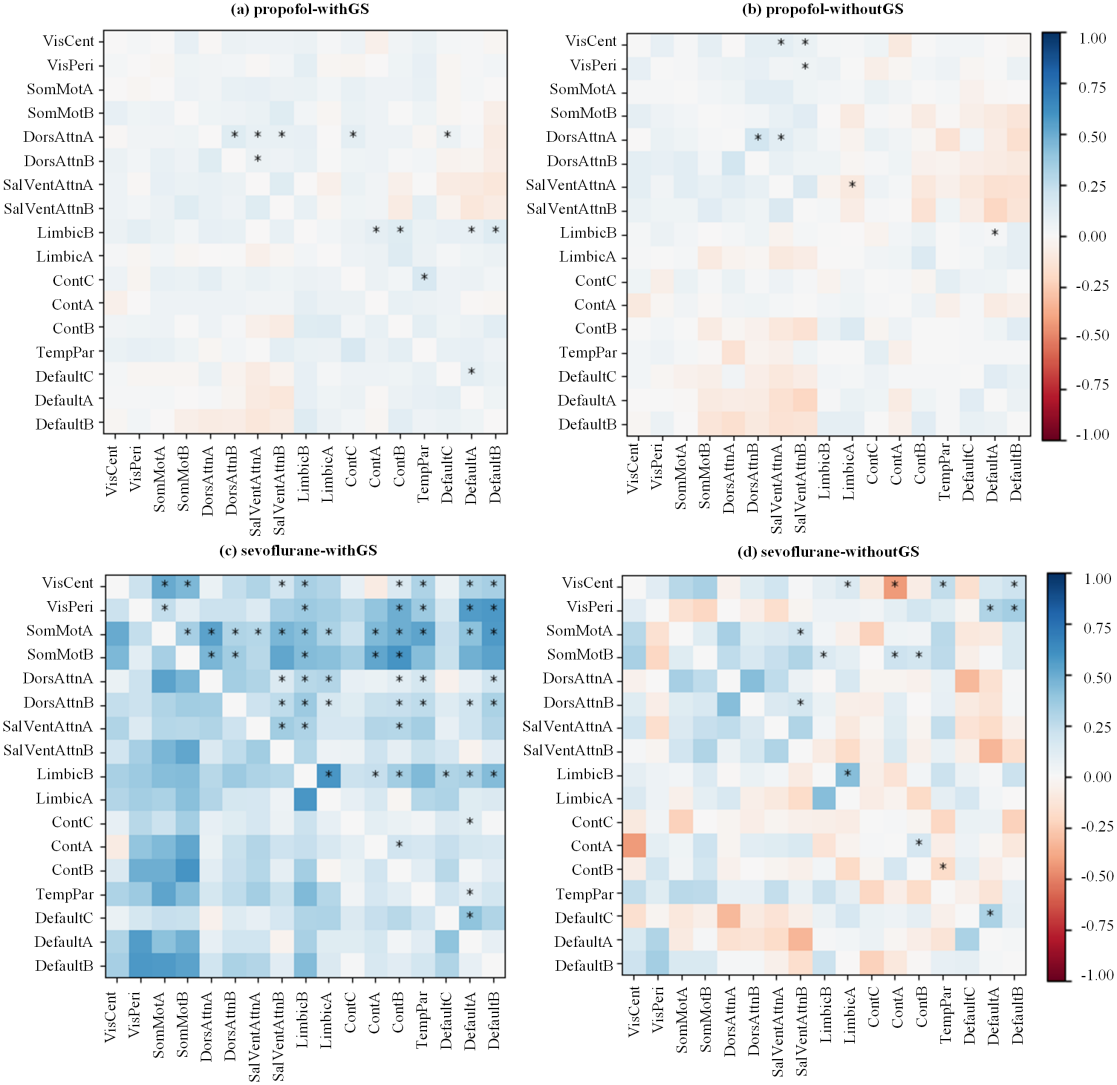
Anesthetic-specific changes in functional connectivity matrices following GSR. The state differences of FC matrix from baseline to unconscious state are calculated by mean baseline Pearson’s coefficient minus mean unconscious state value (red for decreasing and blue for increasing). All the Pearson’s coefficients were then Fisher’s Z transformed. The transformed values were applied Bayesian paired samples t tests (two-tailed). The significances are annotated in the upper triangle of the matrix (*BF_10_ > 3 between the states).

Considering two different conditions (i.e., withGS and withoutGS), to investigate the effect of GSR across consciousness states, we quantified FC changes between baseline and unconscious states for each anesthetic agent. Figure 3 illustrates the differential FC patterns, calculated as the difference between mean baseline and unconscious state FC values. The predominantly blue coloration indicates widespread decreases in network FC from baseline to unconscious states. The upper triangle marked the statistical significances between different states (using the black star; *BF_10_ > 3 between states).

In the withGS propofol condition (Figure 3a), FC predominantly decreased following LOC. This decrease was most pronounced in the Dorsal Attention Network A and Limbic Network B. In the withoutGS propofol condition (Figure 3b), compared with the withGS condition, some decreased FC showed reversals and became increased FC. (e.g., the FC between Control network B and Somatomotor network B) in withoutGS condition and the significant changes focused on Visual networks and Dorsal Attention network A. Within-network FC, particularly between subnetworks of Dorsal Attention Networks A and B, showed significant decreases post-LOC in both conditions (BF_10_ = 9.74, 0.20 [CI: 0.044, 0.330] and BF_10_ = 6.07, 0.233 [CI: 0.038, 0.415], respectively).

During sevoflurane anesthesia with GS (Figure 3c), most FC decreased significantly after LOC, particularly in Somatomotor networks and Limbic network B. In contrast, sevoflurane anesthesia without GS showed fewer decreases in FC after LOC (Figure 3d). Some FC patterns even showed an increasing trend after LOC. Excluding Visual networks, significantly reduced FC after LOC was primarily observed between Somatomotor networks A and Limbic network B (BF_10_ = 3.31, 0.156 [CI: 0.003, 0.306]), as well as between Somatomotor network B and Control networks (A and B; BF_10_ = 10.21, 0.199 [CI: 0.045, 0.325] and BF_10_ = 8.043, 0.168 [CI: 0.044, 0.280], respectively). Additionally, regarding within-network FC in both GS and non-GS conditions, the FC reduction in Limbic network (BF_10_ = 11.74, 0.375 [CI: 0.107, 0.609] and BF_10_ = 26.54, 0.308 [CI: 0.126, 0.446], respectively), Control networks (A and B; BF_10_ = 29.65, 0.184 [CI: 0.074, 0.268] and BF_10_ = 3.46, 0.135 [CI: 0.003, 0.256], respectively) and Default networks (A and C; BF_10_ = 8.87, 0.255 [CI: 0.066, 0.429] and BF_10_ = 8.96, 0.262 [CI: 0.068, 0.439], respectively) exhibited statistical significance.

Further analysis of the FC values revealed that under withGS conditions, both propofol and sevoflurane demonstrated positive FC in baseline and unconscious states, whereas conditions without GS revealed negative between-network FC (Supplementary Figure S4), providing additional context for the observed FC changes. Overall, regarding functional connectivity at the network level from wakefulness to anesthesia, the application of GSR reduced the number of significantly affected networks from 12 to 7 for propofol, and from 57 to 15 for sevoflurane. The application of GSR altered both the quantity and distribution of functional connections that exhibited significant changes during the transition from wakefulness to anesthesia.

### 3.3 Differential impact of global signal on brain network topology during anesthesia

**Figure 4:**
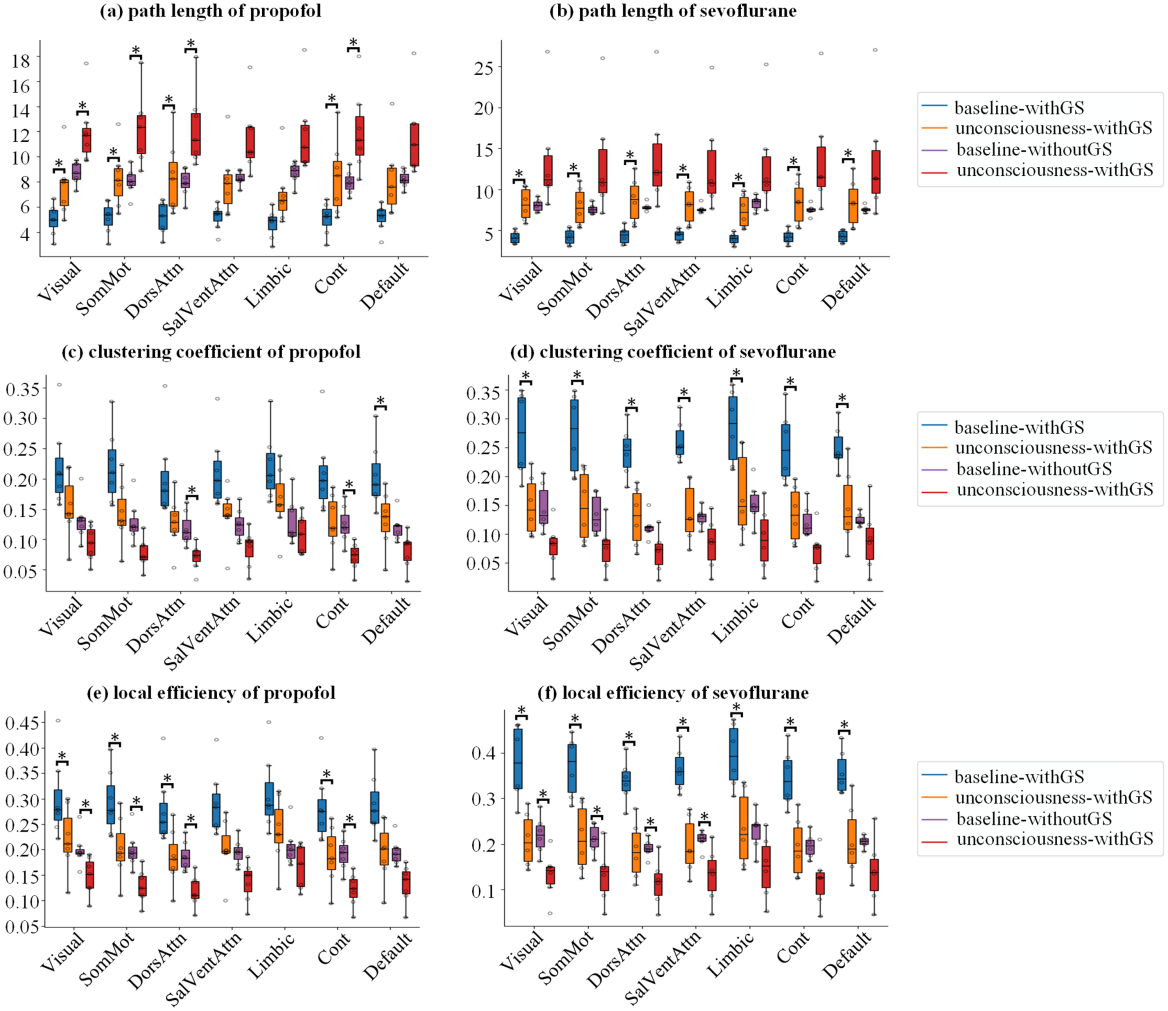
Network topology alterations in nodal-level metrics during anesthesia. Group statistic boxplot of path length **(a,b)**, clustering coefficient **(c,d)** and local efficiency **(e,f)** from baseline to unconscious state for propofol and sevoflurane, respectively, in which the subject level value derived by averaging the ROIs of the corresponding networks. The asterisks refer to significance level (*BF_10_ > 3 between the states) for the Bayesian paired samples t tests.

In present study, we analyzed the influence of GS on brain network indices using graph theory in different anesthetics-induced general anesthesia. We analyzed five brain network indices: path length (Equations 1, 2), clustering coefficient (Equations 3, 4), local efficiency (Equation 5), global efficiency (Equation 6), and small worldness (Equation 7). The first three indices represent nodal-level measurements, while the last two characterize global-level properties. Figure 4 demonstrated the results of path length, clustering coefficient and local efficiency in 7 networks, which were obtained by averaging the ROIs of the corresponding networks at subject level.

For the nodal indices, we first analyzed the interactions of states (baseline and unconscious state), GS conditions (withGS and withoutGS) and network (17 brain networks to which 114 ROIs belong). For the three nodal indices of propofol anesthesia, no significant three-way interaction effect or two-way interaction effect between indices was found. Similarly, under sevoflurane anesthesia, no significant three-way interaction was observed between state and GS condition for any nodal indices (pd < 97.5%). However, we did find a significant two-way interaction between states and GS condition in local efficiency (pd = 97.89%). To further investigate this finding, we conducted Bayesian ANOVA analysis to determine the individual contributions of these factors. The analysis yielded BF_10_(state) = 1.672e+39 and BF_10_(GS condition) = 2.162e+36, indicating that consciousness state was the primary factor influencing local efficiency changes under sevoflurane anesthesia.

For path length during propofol anesthesia (Figure 4a; Table 1) withGS, we found that the path length significantly increased after LOC in Visual, SomMot, DorsAttn and Cont networks networks. Similarly, in the withoutGS condition, significant increases of path length were found in Visual, SomMot, DorsAttn and Cont network networks. For path length under sevoflurane anesthesia, as shown in Figure 4b, path length significantly increased with GS after LOC, in all networks. However, no significant changes occurred after LOC in the without-GS condition.

**Table 1:** The Bayes factor (BF_10_) for the Bayesian paired t-test of path length before and after anesthesia was calculated for both propofol and sevoflurane anesthesia under two conditions: withGS and withoutGS.

Network	Propofol		Sevoflurane	
	withGS	withoutGS	withGS	withoutGS
Visual	3.12*	4.40*	17.59*	1.60
SomMot	4.39*	4.87*	8.28*	1.51
DorsAttn	3.02*	4.94*	7.99*	1.87
SalVentAttn	2.99	2.83	6.38*	1.66
Limbic	2.84	2.10	8.84*	1.26
Cont	3.07*	4.63*	6.32*	1.92
Default	2.74	2.39	3.93*	1.39

Values with BF_10_ > 3 are marked with an asterisk (*), indicating significant changes from baseline to unconsciousness.

Figures 4c,d show the statistics of clustering coefficients in 7 networks. From baseline to unconscious state, propofol administration with GS led to significant decreases in clustering coefficients in DorsAttn network. On the other hand, propofol administration without GS resulted in significant decreases in clustering coefficients in DorsAttn and Cont networks (Figure 4c; Table 2). During sevoflurane anesthesia, clustering coefficients with GS exhibited significant decreases in all networks (Figure 4d). However, in the condition without GS, significant decreases in clustering coefficient from baseline to unconscious state were not observed in any network (Table 2).

**Table 2:** The Bayes factor (BF_10_) for the Bayesian paired t-test of clustering coefficient before and after anesthesia was calculated for both propofol and sevoflurane anesthesia under two conditions: withGS and withoutGS.

Network	Propofol		Sevoflurane	
	withGS	withoutGS	withGS	withoutGS
Visual	1.32	1.08	6.43*	2.39
SomMot	2.29	2.66	7.06*	2.39
DorsAttn	1.63	3.38*	10.61*	2.33
SalVentAttn	2.25	1.37	14.52*	1.69
Limbic	1.55	0.55	3.59*	1.62
Cont	2.34	4.59*	4.64*	1.64
Default	3.07*	2.04	3.70*	0.73

Values with BF_10_ > 3 are marked with an asterisk (*), indicating significant changes from baseline to unconsciousness.

The group statistics of local efficiency were demonstrated in Figures 4e,f. Comparing with baseline, local efficiency significantly decreased in Visual, SomMot, DorsAttn, and Cont networks (Figure 4e) in unconscious state induced by propofol withGS. In conditions without GS, similarly, significant reductions of local efficiency were found in Visual, SomMot, DorsAttn, and Cont networks (Table 3). During sevoflurane anesthesia, the results in Figure 4f showed significant decreases in local efficiency in all networks with GS, while significant differences were observed in Visual, SomMot, DorsAttn and SalVentAttn networks without GS (Table 3).

**Table 3:** The Bayes factor (BF_10_) for the Bayesian paired t-test of local efficiency before and after anesthesia was calculated for both propofol and sevoflurane anesthesia under two conditions: withGS and withoutGS.

Network	Propofol		Sevoflurane	
	withGS	withoutGS	withGS	withoutGS
Visual	3.12*	4.40*	13.13*	4.44*
SomMot	4.39*	4.87*	10.17*	3.73*
DorsAttn	3.02*	4.94*	14.34*	5.03*
SalVentAttn	2.99	2.83	22.20*	3.98*
Limbic	2.84	2.10	7.24*	2.29
Cont	3.07*	4.63*	6.54*	2.80
Default	2.74	2.39	6.86*	1.52

Values with BF_10_ > 3 are marked with an asterisk (*), indicating significant changes from baseline to unconsciousness.

Further, we analyzed the differences of three nodal level indices in 114 ROIs between baseline and unconscious state under administration of propofol and sevoflurane. Bayesian linear mixed models (LMMs) were used to analyze the interactions between consciousness state (baseline and unconscious), anesthetic type (propofol and sevoflurane), and GS condition (withGS and withoutGS) for the global indices of network efficiency and small worldness. No significant three-way interactions were observed among the factors for either global index. A significant two-way interaction was detected between consciousness state and GS condition for small worldness (pd = 99.96%). Subsequent Bayesian ANOVA revealed Bayes factors of BF_10_(state) = 0.298 and BF_10_(GS condition) = 1.381e+16 for GS condition, indicating that the GS condition substantially influenced small worldness measures.

**Figure 5:**
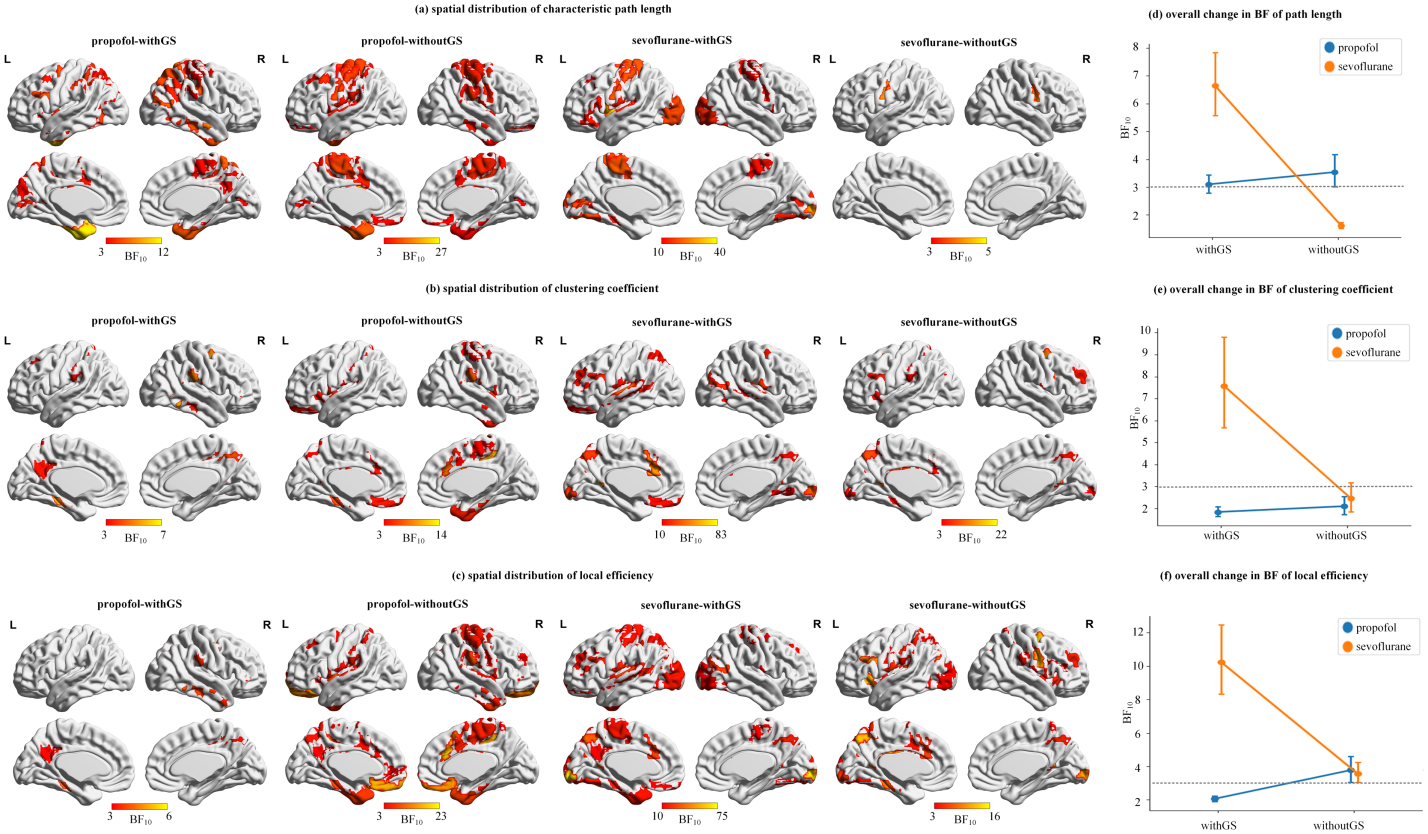
Spatial distribution and statistical significance of network topology changes. **(a–c)** are the spatial distributions of path length, clustering coefficient and local efficiency, which shows the ROIs that have significant changes from baseline to unconscious state (BF_10_ > 3) and with higher BF_10_ got closer to yellow. The color bar indicates the BF_10_ of states differences from baseline to unconscious state. **(d–f)** are the degree of whole brain statistical significance (i.e., mean and SD) of the 114 ROIs BF_10_ in path length, clustering coefficient and local efficiency. The gray dashed line indicated the significant threshold BF_10_ = 3.

Figure 5 demonstrated the spatial distribution of significance of three nodal level indices. The left part of Figure 5 showed the ROIs that have significant changes from baseline to unconscious state (BF_10_ > 3), which with higher BF_10_ got closer to yellow. The right part of Figure 5 showed the mean and SD of all the BF_10_ in the condition of withGS or withoutGS. The gray dashed line indicated the significant threshold BF_10_ = 3. As shown in Figures 5a–c, for three nodal level indices of propofol and sevoflurane, removing GS changed the significance of brain regions (e.g., path length of withGS propofol did not showed significance in left precentral gyrus but in the condition of withoutGS showed significance). The removal of GS led to an increase in the number of significant regions under propofol administration, whereas under sevoflurane, the spatial distribution of significant regions decreased. As illustrated in Figures 5d–f, the removal of GS marginally enhanced the overall significance of nodal indices under propofol administration, while it resulted in reduced significance under sevoflurane.

**Figure 6:**
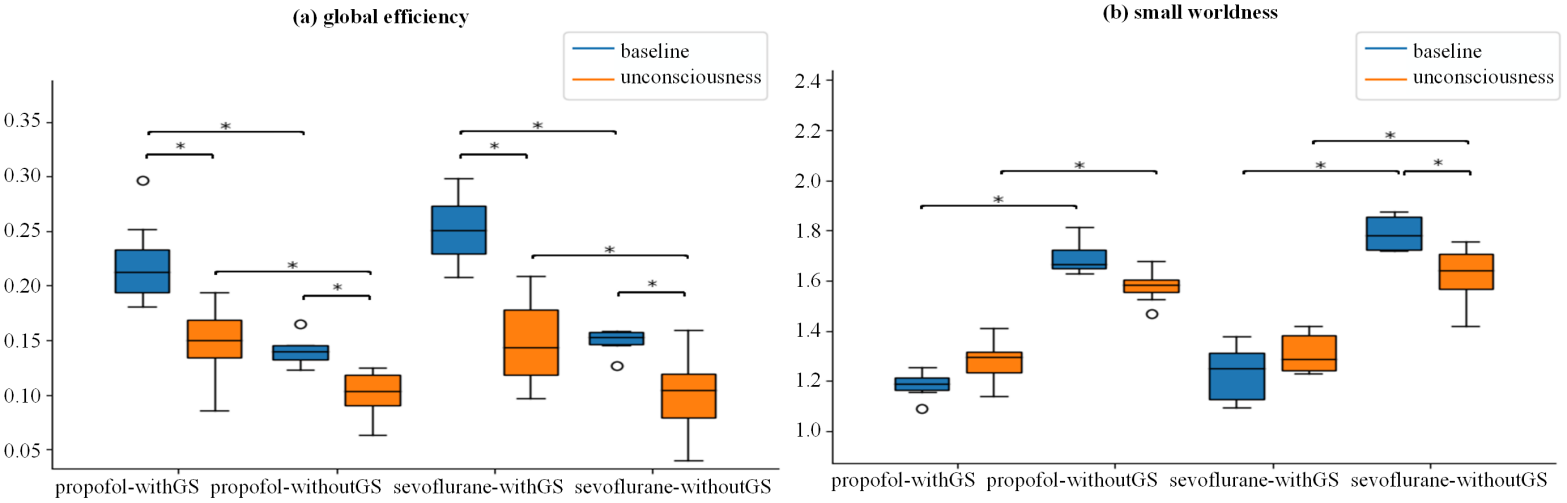
Global network properties during anesthetic-induced unconsciousness. Figure 6 **(a)**-**(b)** are the group statistic boxplots of global efficiency and small worldness from baseline to unconscious state respectively in the condition of withGS and withoutGS. The asterisks refer to significance level (*BF_10_ > 3 between the states) for the Bayesian paired samples *t* tests (two-tailed). WithGS: data processed retaining GS; withoutGS: data processed removing GS.

Figure 6a and Supplementary Tables S20, S21 presents the global efficiency distributions for propofol and sevoflurane as boxplots. Both anesthetics demonstrated significant decreases in global efficiency from baseline to unconscious state under both withGS (propofol: BF_10_ = 2.53, 0.091 [CI: 0.039, 0.140]; sevoflurane: BF_10_ = 10.64, 0.157 [CI: 0.104, 0.213]) and withoutGS (propofol: BF_10_ = 4.02, 0.063 [CI: 0.010, 0.113]; sevoflurane: BF_10_ = 3.02, 0.075 [CI: 0.019, 0.129]). GS removal significantly reduced global efficiency values for both propofol (baseline: BF_10_ = 9.70, 0.095 [CI: 0.045, 0.146]; unconscious state: BF_10_ = 41.37, 0.067 [CI: 0.016, 0.117]) and sevoflurane (baseline: BF_10_ = 84.41, 0.153 [CI: 0.099, 0.206]; unconscious state: BF_10_ = 82.34, 0.07 [CI: 0.016, 0.125]).

Figure 6b and Supplementary Tables S22, S23 showed that small worldness showed a significant decrease only in the withoutGS condition with sevoflurane (BF_10_ = 5.39, 0.167 [CI: 0.070, 0.258]). GS removal significantly increased small worldness for both propofol (baseline: BF_10_ = 1.26e+05, −0.51 [CI: −0.602, −0.427]; unconscious state: BF_10_ = 855.629, −0.3 [CI: −0.386, −0.211]) and sevoflurane (baseline: BF_10_ = 104.62, −0.556 [CI: −0.655, −0.462]; unconscious state: BF_10_ = 94.26, −0.31 [CI: −0.404, −0.212]). Both anesthetics showed trends toward increased small worldness in the withGS condition but decreased small worldness in the withoutGS condition. Based on the equation of small worldness, we found that the divergent trends between withGS and withoutGS conditions were primarily attributable to changes in the normalized clustering coefficient rather than the normalized characteristic path length (Supplementary Figure S5; Supplementary Tables S24–S27).

## 4 Discussion

### 4.1 Preserved state-dependent temporal dynamics following global signal regression

fMRI provides an indirect measure of neural activity through BOLD signals in brain voxels across the temporal domain. Higher SD in these signals indicates a greater dynamic range of brain activity in response to stimuli (Garrett et al., 2013). The overall brain activity level can be assessed by averaging time series data across the entire brain. In the present study, we found that the whole brain fluctuation (i.e., SD of GM average time series) differentiated between conscious states, both with and without GSR under both anesthetic conditions. While GSR influenced the BOLD data in both baseline and unconscious states, its impact on the differences between states was not pronounced (Figure 2a). Notably, voxel-wise SD showed no significant differences between conditions with and without GSR.

Previous research has demonstrated significant relationships between global signal regression and cognitive function. Zhang et al. demonstrated that the SD of GM average time series with GSR correlates with cognitive function (Zhang et al., 2022), while Tanabe et al. reported statistically significant differences in the SD of GM average time series across various states of consciousness (Tanabe et al., 2020). Our findings complement these studies by showing that GSR minimally affects temporal domain analysis indices during general anesthesia, contributing to our understanding of global signal effects in anesthetic contexts.

In terms of frequency analysis, ALFF serves as an effective voxel-wise index for detecting low-frequency oscillations (LFO) near major vessels (Zou et al., 2008). General anesthetics are known to influence cerebral blood flow both directly and indirectly (Slupe and Kirsch, 2018). Given that general anesthesia suppresses neural activity, we hypothesized that this suppression would be detectable in ALFF measurements.

Our results revealed that both propofol and sevoflurane administration showed a trend toward decreased ALFF from baseline to unconscious states (Figure 2c), though this decrease reached statistical significance only for propofol in withoutGS condition. The significant ALFF decrease observed specifically in withoutGS condition for propofol suggests that GSR may remove certain neural signals that are particularly sensitive to action mechanism of propofol. Nevertheless, the consistent downward pattern in ALFF values across both anesthetics suggests that GSR minimally affects the directional pattern of ALFF changes between consciousness states. Furthermore, consistent with previous studies on propofol-induced general anesthesia (Liu X. et al., 2017), both anesthetic agents demonstrated reduced ALFF in regions associated with higher cognitive functions, including the cingulate gyrus, bilateral precentral gyrus, insula, and hippocampus under GSR conditions (Supplementary Figures S1, S2). The involvement of these brain regions, known for their roles in higher cognitive processing (Rolls, 2019; Menon and Uddin, 2010; Bird and Burgess, 2008), aligns with the functional effects of anesthesia and emphasizes the potential utility of GS analysis in monitoring consciousness states.

### 4.2 Anesthetic-specific modulation of brain network organization by global signal regression

The GS in fMRI has been shown to have significant physiological implications, whether retained or removed (Fox et al., 2009; Li et al., 2019; Qing et al., 2015; Wang et al., 2015; Murphy and Fox, 2017; Gotts et al., 2013). Following Yeo et al. (2015), withGS FC represents the widespread information transition throughout the whole brain, indicating global information spread. Conversely, withoutGS FC reflects potential intrinsic connection, suggesting the information exchanged regionally.

In propofol-induced anesthesia, the hippocampus and insula, critical structures within the limbic network (Huang et al., 2021; Pryor et al., 2015), are implicated in cognitive processing during general anesthesia. FC analysis with retained GS under propofol administration revealed significant decreases in connectivity between the Limbic network and Default network, as well as between the Limbic network and Control network. Notably, the withGS state differences under propofol were less pronounced compared to sevoflurane (Figures 3a,c). During sevoflurane anesthesia with retained GS, the transition from baseline to unconscious state was characterized by widespread significant FC decreases. This finding aligns with previous research demonstrating sevoflurane-induced reduction in temporal correlations within the motor cortex (Peltier et al., 2005), consistent with our observation of significantly reduced within-network FC between Somatomotor networks A and B in the withGS condition (Figure 3c). GS removal eliminated the statistical significance of connectivity between the Limbic and Default networks in propofol-induced anesthesia, suggesting GS’s substantial contribution to FC between these regions. For sevoflurane, GS removal resulted in pronounced decreases across most FCs, indicating a substantial global signal effect. These findings suggest that sevoflurane induces more extensive global hemodynamic signal changes than propofol, highlighting the differential role of GS across anesthetic agents.

Previous research indicates that propofol and sevoflurane have different effects on regional cerebral blood flow (rCBF) and the metabolic rate of oxygen (rCMRO2). Propofol comparably reduces both parameters, while sevoflurane has a lesser impact on rCBF with similar effects on rCMRO2 (Kaisti et al., 2003). Our results demonstrate more pronounced FC changes in sevoflurane-induced anesthesia compared to propofol (Figure 3), suggesting that rCBF alterations are not the primary mechanism underlying sevoflurane’s widespread FC decreases. This observation indicates that the differential effects on functional connectivity likely arise from factors beyond simple vascular modulation.

While our study cannot directly establish causality, these differences in FC patterns may potentially relate to the distinct receptor mechanisms of these anesthetics. Propofol primarily modulates *γ*-aminobutyric acid type A (GABAA) receptors (Hales and Lambert, 1991), whereas sevoflurane affects multiple receptor systems including GABAA, N-methyl-D-aspartate (NMDA), and nicotinic acetylcholine (ACh) receptors (Palanca et al., 2017). These pharmacological differences could theoretically contribute to the observed variations in FC patterns through their differential effects on neural signaling across brain regions. However, further research combining pharmacological interventions with neuroimaging would be needed to establish a direct mechanistic link.

Graph theoretical analysis revealed consistent changes across both anesthetics, including increased path length and decreased clustering coefficient and local efficiency across different networks during the transition from baseline to unconscious state (Figure 4). These changes in nodal-level indices under both withGS and withoutGS conditions reflect alterations in network topology and functional network disconnections, which aligns with previous findings (Lee and Mashour, 2018). However, the effects of GSR showed anesthetic-specific patterns. For propofol, GSR demonstrated differential effects on nodal-level indices across functional networks (Figure 4c), in which the network-specific GSR effects indicate that GSR selectively alters the statistical significance of network changes during propofol-induced unconsciousness. As Nalci et al. suggested, GSR functions as a temporal downweighting process, primarily attenuating voxels that significantly contribute to the global signal (Nalci et al., 2017). Our results indicate varying contributions of different networks to the GS during propofol-induced anesthesia. In contrast, sevoflurane anesthesia with GSR led to substantially reduced statistical significance in nodal brain network indices between states across the whole brain (Figure 4), suggesting a fundamental impact of GS on state-related changes. Regarding global-level indices, small worldness group differences increased with retained GS but decreased significantly following GS removal for both anesthetics. Further investigation revealed that the decline in normalized clustering coefficient primarily drove the reduced small worldness following GS removal (Supplementary Figure S4). Previous studies reported increased small-worldness with propofol under withGS conditions (Monti et al., 2013; Schroter et al., 2012), consistent with our findings. We propose that GSR disconnected nodes and reduced clustering coefficients during normalized clustering coefficient calculation, leading to unexpected decreases in small-worldness across states.

While GSR effectively removes nuisance confounds such as head motion and respiration (Power et al., 2014; Liu T. T. et al., 2017). Studies have demonstrated associations between GS and local field potential (Scholvinck et al., 2010). These findings suggest that GS may not merely represent confounding components but may be integral to the brain’s information-dissemination processes (Tanabe et al., 2020). Considering Murphy et al.’s observation that GSR can differentially affect groups and brain networks (Murphy and Fox, 2017), combined with our findings showing that GS removal alters FC patterns and group differences, we recommend retaining GS when analyzing anesthesia-related brain activity, particularly in functional connectivity and network analyses. While GSR minimally affected temporal variability metrics, its substantial impact on network-level measurements suggests that GS contains neural relevant information about brain integration processes essential to consciousness states.

This study has several limitations that need to be addressed. The primary limitation of this study is its relatively small sample size, which may limit the generalizability of our findings. More importantly, our study population consisted of patients with gliomas rather than healthy volunteers, which introduces potential confounding factors. Although we excluded patients with large lesions to minimize the impact of pathology on our results, even smaller tumors could affect neurovascular coupling, regional brain function, and network organization. Additionally, previous research has shown that the widespread GS could result from a common source broadcasting local signals to the whole brain (Turchi et al., 2018; Liu et al., 2018). However, our study only investigates the cortical changes. Future research should examine thalamus and other subcortical structures to investigate the difference between propofol and sevoflurane.

## 5 Conclusion

The primary goal of this study was to investigate how GSR affects the analysis of brain activity during anesthetic-induced unconsciousness, and whether these effects differ between propofol and sevoflurane anesthesia. Our comprehensive analysis of temporal variability indices, ALFF, functional connectivity, and graph theoretical metrics reveals several key findings. We demonstrated that while GSR has minimal effects on temporal and frequency analysis metrics, and it significantly impacts functional connectivity and graph theoretical analyses during general anesthesia, with particularly pronounced effects in sevoflurane-induced unconsciousness. As GSR selectively alters network connections under propofol anesthesia but broadly diminishes connectivity differences under sevoflurane, the anesthetic-specific nature of these effects suggests that careful consideration should be given to GSR application when investigating anesthetic-induced unconsciousness using fMRI, especially when comparing different anesthetic agents.

These findings provide important methodological insights for future research examining consciousness mechanisms and the role of global brain signals in anesthetic-induced unconsciousness. We recommend retaining the GS when comparing different anesthetic agents in fMRI studies, as GSR can obscure genuine agent-specific effects on brain networks. For sevoflurane studies in particular, removing GS substantially reduces the statistical significance of network alterations during unconsciousness, potentially leading to underestimation of network disruption. Rather than treating GS as mere noise, future consciousness research should carefully consider anesthetic-specific GS effects in anesthesia, particularly for sevoflurane, where GS appears to carry substantial information about state-dependent network reorganization.

## Data Availability

The data analyzed in this study is subject to the following licenses/restrictions: the dataset contains sensitive patient information and cannot be publicly shared. However, the data may be available upon reasonable request from qualified researchers, subject to appropriate institutional approval and data protection requirements. Requests to access these datasets should be directed to Zhenhu Liang, zhl@ysu.edu.cn.

## Footnotes

^1^ https://github.com/aestrivex/bctpy

^2^ http://www.r-project.org

## References

[ref1] Alkire M. T. Hudetz A. G. Tononi G. (2008). Consciousness and anesthesia. Science 322, 876–880. doi: 10.1126/science.1149213, PMID: 18988836 PMC2743249

[ref2] Almgren H. Van de Steen F. Razi A. Friston K. Marinazzo D. (2020). The effect of global signal regression on DCM estimates of noise and effective connectivity from resting state fMRI. NeuroImage 208:116435. doi: 10.1016/j.neuroimage.2019.116435, PMID: 31816423 PMC7014820

[ref3] Benjamin D. J. Berger J. O. Johannesson M. Nosek B. A. Wagenmakers E. J. Berk R.. (2018). Redefine statistical significance. Nat. Hum. Behav. 2, 6–10. doi: 10.1038/s41562-017-0189-z, PMID: 30980045

[ref4] Bird C. M. Burgess N. (2008). The hippocampus and memory: insights from spatial processing. Nat. Rev. Neurosci. 9, 182–194. doi: 10.1038/nrn2335, PMID: 18270514

[ref5] Blain-Moraes S. Tarnal V. Vanini G. Bel-Behar T. Janke E. Picton P.. (2017). Network efficiency and posterior alpha patterns are markers of recovery from general anesthesia: a high-density electroencephalography study in healthy volunteers. Front. Hum. Neurosci. 11:328. doi: 10.3389/fnhum.2017.00328, PMID: 28701933 PMC5487412

[ref6] Brown E. N. Lydic R. Schiff N. D. (2010). General anesthesia, sleep, and coma. N. Engl. J. Med. 363, 2638–2650. doi: 10.1056/NEJMra0808281, PMID: 21190458 PMC3162622

[ref7] Bürkner P.-C. (2017). Brms: an R package for Bayesian multilevel models using Stan. J. Stat. Softw. 80, 1–28. doi: 10.18637/jss.v080.i01

[ref8] Esteban O. Markiewicz C. J. Blair R. W. Moodie C. A. Isik A. I. Erramuzpe A.. (2019). fMRIPrep: a robust preprocessing pipeline for functional MRI. Nat. Methods 16, 111–116. doi: 10.1038/s41592-018-0235-4, PMID: 30532080 PMC6319393

[ref18] Etz A. Vandekerckhove J. (2016). Bayesian Perspective on the Reproducibility Project: Psychology. PLoS One 11:e0149794. doi: 10.1371/journal.pone.0149794, PMID: 26919473 PMC4769355

[ref9] Fox M. D. Zhang D. Snyder A. Z. Raichle M. E. (2009). The global signal and observed anticorrelated resting state brain networks. J. Neurophysiol. 101, 3270–3283. doi: 10.1152/jn.90777.2008, PMID: 19339462 PMC2694109

[ref10] Friston K. J. (1994). Functional and effective connectivity in neuroimaging: a synthesis. Hum. Brain Mapp. 2, 56–78.

[ref11] Garrett D. D. Samanez-Larkin G. R. MacDonald S. W. Lindenberger U. McIntosh A. R. Grady C. L. (2013). Moment-to-moment brain signal variability: a next frontier in human brain mapping? Neurosci. Biobehav. Rev. 37, 610–624. doi: 10.1016/j.neubiorev.2013.02.015, PMID: 23458776 PMC3732213

[ref12] Gotts S. J. Saad Z. S. Jo H. J. Wallace G. L. Cox R. W. Martin A. (2013). The perils of global signal regression for group comparisons: a case study of autism Spectrum disorders. Front. Hum. Neurosci. 7:356. doi: 10.3389/fnhum.2013.00356, PMID: 23874279 PMC3709423

[ref13] Hales T. G. Lambert J. J. (1991). The actions of propofol on inhibitory amino acid receptors of bovine adrenomedullary chromaffin cells and rodent central neurones. Br. J. Pharmacol. 104, 619–628. 1665745 10.1111/j.1476-5381.1991.tb12479.x PMC1908220

[ref14] Hillman E. M. (2014). Coupling mechanism and significance of the BOLD signal: a status report. Annu. Rev. Neurosci. 37, 161–181. doi: 10.1146/annurev-neuro-071013-014111, PMID: 25032494 PMC4147398

[ref15] Huang Z. Tarnal V. Vlisides P. E. Janke E. L. McKinney A. M. Picton P.. (2021). Anterior insula regulates brain network transitions that gate conscious access. Cell Rep. 35:109081. doi: 10.1016/j.celrep.2021.109081, PMID: 33951427 PMC8157795

[ref16] Huang Z. Vlisides P. E. Tarnal V. C. Janke E. L. Keefe K. M. Collins M. M.. (2018). Brain imaging reveals covert consciousness during behavioral unresponsiveness induced by propofol. Sci. Rep. 8:13195. doi: 10.1038/s41598-018-31436-z, PMID: 30181567 PMC6123455

[ref17] Huang Z. Zhang J. Wu J. Qin P. Wu X. Wang Z.. (2016). Decoupled temporal variability and signal synchronization of spontaneous brain activity in loss of consciousness: an fMRI study in anesthesia. NeuroImage 124, 693–703. doi: 10.1016/j.neuroimage.2015.08.062, PMID: 26343319

[ref19] Kaisti K. K. Langsjo J. W. Aalto S. Oikonen V. Sipila H. Teras M.. (2003). Effects of sevoflurane, propofol, and adjunct nitrous oxide on regional cerebral blood flow, oxygen consumption, and blood volume in humans. Anesthesiology 99, 603–613. doi: 10.1097/00000542-200309000-00015, PMID: 12960544

[ref20] Larson C. Kaplan D. Girolamo T. (2023). A Bayesian statistics tutorial for clinical research: Prior distributions and meaningful results for small clinical samples. J. Clin. Psychol. 79, 2602–2624. doi: 10.1002/jclp.23570 37477577 PMC10591806

[ref21] Lee J.-M. Kim P.-J. Kim H.-G. Hyun H.-K. Kim Y. J. Kim J.-W.. (2020). Analysis of brain connectivity during nitrous oxide sedation using graph theory. Sci. Rep. 10:2354. doi: 10.1038/s41598-020-59264-0, PMID: 32047246 PMC7012909

[ref22] Lee U. Mashour G. A. (2018). Role of network science in the study of anesthetic state transitions. Anesthesiology 129, 1029–1044. doi: 10.1097/ALN.0000000000002228, PMID: 29683806 PMC6191341

[ref23] Li J. Kong R. Liegeois R. Orban C. Tan Y. Sun N.. (2019). Global signal regression strengthens association between resting-state functional connectivity and behavior. NeuroImage 196, 126–141. doi: 10.1016/j.neuroimage.2019.04.016, PMID: 30974241 PMC6585462

[ref24] Li Y. Li F. Zheng H. Jiang L. Peng Y. Zhang Y.. (2021). Recognition of general anesthesia-induced loss of consciousness based on the spatial pattern of the brain networks. J. Neural Eng. 18:056039. doi: 10.1088/1741-2552/ac27fc, PMID: 34534980

[ref25] Liu X. de Zwart J. A. Scholvinck M. L. Chang C. Ye F. Q. Leopold D. A.. (2018). Subcortical evidence for a contribution of arousal to fMRI studies of brain activity. Nat. Commun. 9:395. doi: 10.1038/s41467-017-02815-3, PMID: 29374172 PMC5786066

[ref26] Liu X. Lauer K. K. Ward B. D. Roberts C. Liu S. Gollapudy S.. (2017). Propofol attenuates low-frequency fluctuations of resting-state fMRI BOLD signal in the anterior frontal cortex upon loss of consciousness. NeuroImage 147, 295–301. doi: 10.1016/j.neuroimage.2016.12.043, PMID: 27993673 PMC5303656

[ref27] Liu T. T. Nalci A. Falahpour M. (2017). The global signal in fMRI: nuisance or information? NeuroImage 150, 213–229. doi: 10.1016/j.neuroimage.2017.02.036, PMID: 28213118 PMC5406229

[ref28] Makowski D. Ben-Shachar M. S. Chen S. H. A. Ludecke D. (2019). Indices of effect existence and significance in the Bayesian framework. Front. Psychol. 10:2767. doi: 10.3389/fpsyg.2019.02767, PMID: 31920819 PMC6914840

[ref29] Menon V. Uddin L. Q. (2010). Saliency, switching, attention and control: a network model of insula function. Brain Struct. Funct. 214, 655–667. doi: 10.1007/s00429-010-0262-0, PMID: 20512370 PMC2899886

[ref30] Monti M. M. Lutkenhoff E. S. Rubinov M. Boveroux P. Vanhaudenhuyse A. Gosseries O.. (2013). Dynamic change of global and local information processing in propofol-induced loss and recovery of consciousness. PLoS Comput. Biol. 9:e1003271. doi: 10.1371/journal.pcbi.1003271, PMID: 24146606 PMC3798283

[ref31] Murphy K. Fox M. D. (2017). Towards a consensus regarding global signal regression for resting state functional connectivity MRI. NeuroImage 154, 169–173. doi: 10.1016/j.neuroimage.2016.11.052, PMID: 27888059 PMC5489207

[ref32] Nalci A. Rao B. D. Liu T. T. (2017). Global signal regression acts as a temporal downweighting process in resting-state fMRI. NeuroImage 152, 602–618. doi: 10.1016/j.neuroimage.2017.01.015, PMID: 28089677

[ref33] Palanca B. J. A. Avidan M. S. Mashour G. A. (2017). Human neural correlates of sevoflurane-induced unconsciousness. Br. J. Anaesth. 119, 573–582. doi: 10.1093/bja/aex244, PMID: 29121298 PMC6172973

[ref34] Palanca B. J. Mitra A. Larson-Prior L. Snyder A. Z. Avidan M. S. Raichle M. E. (2015). Resting-state functional magnetic resonance imaging correlates of sevoflurane-induced unconsciousness. Anesthesiology 123, 346–356. doi: 10.1097/ALN.0000000000000731, PMID: 26057259 PMC4509973

[ref35] Peltier S. J. Kerssens C. Hamann S. B. Sebel P. S. Byas-Smith M. Hu X. (2005). Functional connectivity changes with concentration of sevoflurane anesthesia. Neuroreport 16, 285–288. doi: 10.1097/00001756-200502280-00017, PMID: 15706237

[ref36] Power J. D. Mitra A. Laumann T. O. Snyder A. Z. Schlaggar B. L. Petersen S. E. (2014). Methods to detect, characterize, and remove motion artifact in resting state fMRI. NeuroImage 84, 320–341. doi: 10.1016/j.neuroimage.2013.08.048, PMID: 23994314 PMC3849338

[ref37] Pryor K. O. Root J. C. Mehta M. Stern E. Pan H. Veselis R. A.. (2015). Effect of propofol on the medial temporal lobe emotional memory system: a functional magnetic resonance imaging study in human subjects. Br. J. Anaesth. 115, i104–i113. doi: 10.1093/bja/aev038, PMID: 26174294 PMC4501915

[ref38] Qing Z. Dong Z. Li S. Zang Y. Liu D. (2015). Global signal regression has complex effects on regional homogeneity of resting state fMRI signal. Magn. Reson. Imaging 33, 1306–1313. doi: 10.1016/j.mri.2015.07.011, PMID: 26234499

[ref39] Rolls E. T. (2019). The cingulate cortex and limbic systems for emotion, action, and memory. Brain Struct. Funct. 224, 3001–3018. doi: 10.1007/s00429-019-01945-2, PMID: 31451898 PMC6875144

[ref40] Saad Z. S. Gotts S. J. Murphy K. Chen G. Jo H. J. Martin A.. (2012). Trouble at rest: how correlation patterns and group differences become distorted after global signal regression. Brain Connect. 2, 25–32. doi: 10.1089/brain.2012.0080, PMID: 22432927 PMC3484684

[ref41] Scholvinck M. L. Maier A. Ye F. Q. Duyn J. H. Leopold D. A. (2010). Neural basis of global resting-state fMRI activity. Proc. Natl. Acad. Sci. USA 107, 10238–10243. doi: 10.1073/pnas.0913110107, PMID: 20439733 PMC2890438

[ref42] Schroter M. S. Spoormaker V. I. Schorer A. Wohlschlager A. Czisch M. Kochs E. F.. (2012). Spatiotemporal reconfiguration of large-scale brain functional networks during propofol-induced loss of consciousness. J. Neurosci. 32, 12832–12840. doi: 10.1523/JNEUROSCI.6046-11.2012, PMID: 22973006 PMC6703804

[ref43] Slupe A. M. Kirsch J. R. (2018). Effects of anesthesia on cerebral blood flow, metabolism, and neuroprotection. J. Cereb. Blood Flow Metab. 38, 2192–2208. doi: 10.1177/0271678X18789273, PMID: 30009645 PMC6282215

[ref44] Stanley M. L. Simpson S. L. Dagenbach D. Lyday R. G. Burdette J. H. Laurienti P. J. (2015). Changes in brain network efficiency and working memory performance in aging. PLoS One 10:e0123950. doi: 10.1371/journal.pone.0123950, PMID: 25875001 PMC4395305

[ref45] Tanabe S. Huang Z. Zhang J. Chen Y. Fogel S. Doyon J.. (2020). Altered global brain signal during physiologic, pharmacologic, and pathologic states of unconsciousness in humans and rats. Anesthesiology 132, 1392–1406. doi: 10.1097/ALN.0000000000003197, PMID: 32205548 PMC8218242

[ref46] Turchi J. Chang C. Ye F. Q. Russ B. E. Yu D. K. Cortes C. R.. (2018). The basal forebrain regulates global resting-state fMRI fluctuations. Neuron 97, 940–952.e4. doi: 10.1016/j.neuron.2018.01.032, PMID: 29398365 PMC5823771

[ref47] Wang J. Khosrowabadi R. Ng K. K. Hong Z. Chong J. S. X. Wang Y.. (2018). Alterations in brain network topology and structural-functional connectome coupling relate to cognitive impairment. Front. Aging Neurosci. 10:404. doi: 10.3389/fnagi.2018.00404, PMID: 30618711 PMC6300727

[ref48] Wang D. Zhou Y. Zhuo C. Qin W. Zhu J. Liu H.. (2015). Altered functional connectivity of the cingulate subregions in schizophrenia. Transl. Psychiatry 5:e575. doi: 10.1038/tp.2015.69, PMID: 26035059 PMC4490280

[ref49] Yeo B. T. Krienen F. M. Sepulcre J. Sabuncu M. R. Lashkari D. Hollinshead M.. (2011). The organization of the human cerebral cortex estimated by intrinsic functional connectivity. J. Neurophysiol. 106, 1125–1165. doi: 10.1152/jn.00338.2011, PMID: 21653723 PMC3174820

[ref50] Yeo B. T. Tandi J. Chee M. W. (2015). Functional connectivity during rested wakefulness predicts vulnerability to sleep deprivation. NeuroImage 111, 147–158. doi: 10.1016/j.neuroimage.2015.02.018, PMID: 25700949

[ref51] Zang Y. F. He Y. Zhu C. Z. Cao Q. J. Sui M. Q. Liang M.. (2007). Altered baseline brain activity in children with ADHD revealed by resting-state functional MRI. Brain Dev. 29, 83–91. doi: 10.1016/j.braindev.2006.07.002, PMID: 16919409

[ref52] Zhang C. Beste C. Prochazkova L. Wang K. Speer S. P. Smidts A.. (2022). Resting-state BOLD signal variability is associated with individual differences in metacontrol. Sci. Rep. 12:18425. doi: 10.1038/s41598-022-21703-5, PMID: 36319653 PMC9626555

[ref53] Zhang J. Northoff G. (2022). Beyond noise to function: reframing the global brain activity and its dynamic topography. Commun Biol 5:1350. doi: 10.1038/s42003-022-04297-6, PMID: 36481785 PMC9732046

[ref54] Zou Q. H. Zhu C. Z. Yang Y. Zuo X. N. Long X. Y. Cao Q. J.. (2008). An improved approach to detection of amplitude of low-frequency fluctuation (ALFF) for resting-state fMRI: fractional ALFF. J. Neurosci. Methods 172, 137–141. doi: 10.1016/j.jneumeth.2008.04.012, PMID: 18501969 PMC3902859

[ref55] Zuo X. N. Di Martino A. Kelly C. Shehzad Z. E. Gee D. G. Klein D. F.. (2010). The oscillating brain: complex and reliable. NeuroImage 49, 1432–1445. doi: 10.1016/j.neuroimage.2009.09.037, PMID: 19782143 PMC2856476

