# Supplementary material for: Distinct effects of global signal regression on brain activity during propofol and sevoflurane anesthesia
Source: Front Neurosci. 2025 May 22;19:1576535. doi: 10.3389/fnins.2025.1576535 (PMC12137232; doi:10.3389/fnins.2025.1576535)
Supplement: Supplementary file 1 [file Data_Sheet_1.pdf]

## Supplementary material of method

### (1) Supplementation of the anesthesia process

All patients in this study underwent surgery for supratentorial glioma, which required intraoperative magnetic resonance imaging (MRI)-assisted tumor resection. During the MRI scan, various physiological measurements were continuously monitored, including the electrocardiogram (ECG), heart rate (HR), pulse oxygen saturation (SpO2), non-invasive blood pressure (NBP), end-tidal carbon dioxide (ETCO2) and bispectral index (BIS). To monitor invasive arterial pressure (IBP), ultrasound-guided radial artery puncture was performed. Additionally, two venous accesses were established in the upper extremities for all patients. During induction of general anesthesia, all patients were treated by intravenous injection of propofol (1.5-2 mg/kg), remifentanil (1-2 μg/kg), and rocuronium (0.6-1.0 mg/kg). Oral tracheal intubation was assisted by video laryngoscope, and followed by mechanical ventilation using an anesthesia machine with oxygen flow 2.0 L/min, tidal volume 6-8 ml/kg, respiratory rate 10-16 times/min to maintain blood oxygen saturation 98% - 100% and ETCO2 35-40 mmHg. Patients were maintained with propofol and remifentanil, or sevoflurane and remifentanil for 30 minutes respectively without additional sedative and analgesic agents (propofol: 1.3 - 3.5 mg/kg, mean = 2.02, SD = 0.83; sevoflurane: 1~2.5 MAC, mean = 1.75, SD = 0.52). The electrodes of BIS were withdrawn if the BIS can be maintained at a stable level between 40 and 60 for more than 10 minutes, and patients were scanned with the same stable effect-site concentration. Intravenous norepinephrine with pump was administered to prevent mean arterial pressure (MAP) from decreasing beyond 20% of the preoperative level during the scan.

### (2) Supplementation of the data preprocessing

Results included in this manuscript come from preprocessing performed using fMRIPrep 21.0.2 (Esteban, Markiewicz, et al. (2018); Esteban, Blair, et al. (2018); RRID:SCR_016216), which is based on Nipype 1.6.1 (K. Gorgolewski et al. (2011); K.

J. Gorgolewski et al. (2018); RRID:SCR_002502).

**a) Anatomical data preprocessing**

A total of 1 T1-weighted (T1w) images were found within the input BIDS dataset.The T1-weighted (T1w) image was corrected for intensity non-uniformity (INU) with N4BiasFieldCorrection (Tustison et al. 2010), distributed with ANTs 2.3.3 (Avants et al. 2008, RRID:SCR_004757), and used as T1w-reference throughout the workflow. The T1w-reference was then skull-stripped with a Nipype implementation of the antsBrainExtraction.sh workflow (from ANTs), using OASIS30ANTs as target template. Brain tissue segmentation of cerebrospinal fluid (CSF), white-matter (WM) and gray-matter (GM) was performed on the brain-extracted T1w using fast (FSL 6.0.5.1:57b01774, RRID:SCR_002823, Zhang, Brady, and Smith 2001). Brain surfaces were reconstructed using recon-all (FreeSurfer 6.0.1, RRID:SCR_001847, Dale, Fischl, and Sereno 1999), and the brain mask estimated previously was refined with a custom variation of the method to reconcile ANTs-derived and FreeSurfer-derived segmentations of the cortical gray-matter of Mindboggle (RRID:SCR_002438, Klein et al. 2017). Volume-based spatial normalization to one standard space (MNI152NLin2009cAsym) was performed through nonlinear registration with antsRegistration (ANTs 2.3.3), using brain-extracted versions of both T1w reference and the T1w template. The following template was selected for spatial normalization: ICBM 152 Nonlinear Asymmetrical template version 2009c [Fonov et al. (2009), RRID:SCR_008796; TemplateFlow ID: MNI152NLin2009cAsym].

**b) Functional data preprocessing**

For each of the 1 BOLD runs found per subject (across all tasks and sessions), the following preprocessing was performed. First, a reference volume and its skull-stripped version were generated using a custom methodology of fMRIPrep. Head-motion parameters with respect to the BOLD reference (transformation matrices, and six corresponding rotation and translation parameters) are estimated before any spatiotemporal filtering using mcflirt (FSL 6.0.5.1:57b01774, Jenkinson et al. 2002). BOLD runs were slice-time corrected to 1.06s (0.5 of slice acquisition range 0s-2.13s) using 3dTshift from AFNI (Cox and Hyde 1997, RRID:SCR_005927). The BOLD

time-series (including slice-timing correction when applied) were resampled onto their original, native space by applying the transforms to correct for head-motion. These resampled BOLD time-series will be referred to as preprocessed BOLD in original space, or just preprocessed BOLD. The BOLD reference was then co-registered to the T1w reference using bbregister (FreeSurfer) which implements boundary-based registration (Greve and Fischl 2009). Co-registration was configured with six degrees of freedom. Several confounding time-series were calculated based on the preprocessed BOLD: framewise displacement (FD), DVARS and three region-wise global signals. FD was computed using two formulations following Power (absolute sum of relative motions, Power et al. (2014)) and Jenkinson (relative root mean square displacement between affines, Jenkinson et al. (2002)). FD and DVARS are calculated for each functional run, both using their implementations in Nipype (following the definitions by Power et al. 2014). The three global signals are extracted within the CSF, the WM, and the whole-brain masks. Additionally, a set of physiological regressors were extracted to allow for component-based noise correction (CompCor, Behzadi et al. 2007). Principal components are estimated after high-pass filtering the preprocessed BOLD time-series (using a discrete cosine filter with 128s cut-off) for the two CompCor variants: temporal (tCompCor) and anatomical (aCompCor). tCompCor components are then calculated from the top 2% variable voxels within the brain mask. For aCompCor, three probabilistic masks (CSF, WM and combined CSF+WM) are generated in anatomical space. The implementation differs from that of Behzadi et al. in that instead of eroding the masks by 2 pixels on BOLD space, the aCompCor masks are subtracted a mask of pixels that likely contain a volume fraction of GM. This mask is obtained by dilating a GM mask extracted from the FreeSurfer's aseg segmentation, and it ensures components are not extracted from voxels containing a minimal fraction of GM. Finally, these masks are resampled into BOLD space and binarized by thresholding at 0.99 (as in the original implementation). Components are also calculated separately within the WM and CSF masks. For each CompCor decomposition, the k components with the largest singular values are retained, such that the retained components' time series are sufficient to explain 50 percent of variance

across the nuisance mask (CSF, WM, combined, or temporal). The remaining components are dropped from consideration. The head-motion estimates calculated in the correction step were also placed within the corresponding confounds file. The confound time series derived from head motion estimates and global signals were expanded with the inclusion of temporal derivatives and quadratic terms for each (Satterthwaite et al. 2013). Frames that exceeded a threshold of 0.5 mm FD or 1.5 standardised DVARS were annotated as motion outliers. The BOLD time-series were resampled into standard space, generating a preprocessed BOLD run in MNI152NLin2009cAsym space. First, a reference volume and its skull-stripped version were generated using a custom methodology of fMRIPrep. All resamplings can be performed with a single interpolation step by composing all the pertinent transformations (i.e. head-motion transform matrices, susceptibility distortion correction when available, and co-registrations to anatomical and output spaces). Gridded (volumetric) resamplings were performed using antsApplyTransforms (ANTs), configured with Lanczos interpolation to minimize the smoothing effects of other kernels (Lanczos 1964). Non-gridded (surface) resamplings were performed using mri_vol2surf (FreeSurfer).

**(3) Specific calculation of graph theoretical indices**

The characteristic path length, which reflects the brain network's capability for integrating information, is defined as the average shortest path length cross all possible pairs of nodes in the network, whereas the shortest path length is defined as the sum of the minimal weights from one node to another:

$$L = \frac{1}{n}\sum_{i \in N} L_i = \frac{1}{n}\sum_{i \in N} \frac{\sum_{j \in N, j \neq i} d_{ij}}{n-1} \quad (1)$$

where $L_i$ is the average distance between node $i$ and all other nodes. The $d_{ij}$ denotes the weighted shortest path length between node $i$ and $j$. Then the characteristic path length $L$ was normalized:

$$\lambda = \frac{L}{L_{rand}} \quad (2)$$

Where $\lambda$ represents normalized path length (NPL) and $L_{rand}$ denotes the weighted characteristic path length of the set of random networks.

Different brain regions can be grouped for specialized information processing, including integration and segregation. Clustering coefficient is a parameter for qualifying the segregation of brain function, which measures the ability of one node interconnect with other nodes. The clustering coefficient of a node is defined as the fraction of triangles around a node and the whole brain's clustering coefficient is equal to the average clustering coefficient across all nodes:

$$C = \frac{1}{n}\sum_{i\in N} C_i = \frac{1}{n}\sum_{i\in N}\frac{2t_i}{k_i\left(k_i - 1\right)} \tag{3}$$

where $C_i$ is the clustering coefficient of node $i$ ($C_i = 0$ for $k_i < 2$), $t_i$ denotes the weighted geometric mean of triangles around a node $i$, and $k_i$ represented the degree of node $i$. Then the clustering coefficient $C$ was normalized:

$$\gamma = \frac{C}{C_{rand}} \tag{4}$$

where the $\gamma$ denotes the normalized clustering coefficient (NCC) and $C_{rand}$ denotes the clustering coefficient of the set of random networks.

Local efficiency is the index for measuring the information transfer of the subgraph induced by the neighbors of the node. Higher local efficiency indicates that the neural information is processed more separately. The local efficiency is defined as the inverse of the shortest average path length of all neighbors of a given node and the whole brain's local efficiency is the average of all nodes:

$$E_{loc} = \frac{1}{2}\sum_{i\in N}\frac{\sum_{j,h\in N, j\neq i}\left(w_{ij}w_{ih}\left[d_{jh}^{w}\left(N_i\right)\right]^{-1}\right)^{1/3}}{k_i\left(k_i - 1\right)} \tag{5}$$

where $E_{loc}$ is the local efficiency of node $i$, $w_{ij}$ is the weight connection between $i$ and $j$ and $d_{jh}$ ($N_i$) is the length of the shortest path between $j$ and $h$ which contains only neighbors of $i$.

The global efficiency is the average inverse shortest path length in the network, since paths between disconnected nodes are assumed to have infinite lengths and

corresponded to zero efficiency:

$$E_{glob} = \frac{1}{n}\sum_{i\in N} E_i = \frac{1}{n}\sum_{i\in N}\frac{\sum_{j\in N, j\neq i} d_{ij}^{-1}}{n-1} \tag{6}$$

where $E_i$ is the efficiency of node *i*.

The small world organization of human brain is one of the most important findings of graph theory. The small worldness exhibits the ability of information segregation and integration with low energy and wiring costs It is defined as the ratio of the normalized clustering coefficient to the normalized path length:

$$SW = \frac{\gamma}{\lambda} = \frac{C / C_{rand}}{L / L_{rand}} \tag{7}$$

A higher small worldness (i.e., a higher clustering coefficient and lower characteristic path length) means the brain network processes information more effectively. A brain network with $SW > 1$ is regarded as having the small-world characteristic. Additionally, to obtain appropriate $L_{rand}$ and $C_{rand}$, for each subject of each state, we generated 100 random networks with the same nodes, edges and degree distribution as the actual network. $L_{rand}$ and $C_{rand}$ were evaluated as the averaged characteristic path length and the averaged clustering coefficient of sets of random networks.

## Supplementary material of results

### (1) Spatial distribution of voxel-wise SD and ALFF

In time domain and frequency domain analysis, subsequently, we investigated whether voxel-wise ALFF and SD changed spatially after GSR. The uncorrected spatial distributions of ALFF and SD were presented in Supplementary Figure S1. In the scenario of withGS, normalized ALFF of both anesthetics showed decrease, mainly concentrated in cingulate gyrus, bilateral precentral gyrus, insular and hippocampus. However, sparse decreases in normalized ALFF were observed in the scenario of withoutGS (see Supplementary Figure S2). The voxel-wise SD of both anesthetics did not exhibit any concentrated voxel region in the scenario of withGS and withoutGS (see

Supplementary Figure S3). However, further extracting the FDR corrected voxel distribution of normalized ALFF and voxel-wise SD, no significant region was found in both indices for propofol and sevoflurane.

**(2) Validation of interaction effects on temporal and low-frequency indices**

The validation results reveled that there is no significant three-way interaction effect among the factors for SD, voxel-wise SD and ALFF (pd < 97.5%). Subsequently, the three two-way interaction effects were analyzed. Similarly, no significant two-way interaction effects were found in any of the three indicators (pd < 97.5%).

**(3) Validation of interaction effects on graph theoretical nodal indices**

For the nodal indices, we first analyzed the interactions of states (baseline and unconscious state), GS scenarios (withGS and withoutGS) and network (17 brain networks to which 114 ROIs belong). For the three nodal indices of propofol anesthesia, no significant three-way interaction effect or two-way interaction effect between indices was found. For sevoflurane anesthesia, there was no significant three-way interaction effect between state and GS scenario (withGS and withoutGS) in three nodal indices (pd < 97.5%). However, we did find a significant two-way interaction between states (baseline and unconscious state) and GS scenario (withGS and withoutGS) in of local efficiency (pd = 97.89%). Further, Bayesian ANOVA analysis was applied to determine the effects of indices on the local efficiency. Considering state, GS scenario, both state and GS scenario as the main effect separately, $BF_{10}$ of each model were calculated. The Bayesian ANOVA results showed that $BF_{10}$(state) = 1.672e+39 and $BF_{10}$(GS scenario) = 2.162e+36, indicating that the sevoflurane results of local efficiency were mainly affected by state. Thus the further investigation of local efficiency was focused on the effects of state not the GS scenario.

**(4) Validation of interaction effects on graph theoretical global indices**

Bayesian linear mixed models (LMMs) were used to analyze the interactions between consciousness state (baseline and unconscious), anesthetic type (propofol and

sevoflurane), and GS condition (withGS and withoutGS) for the global indices of network efficiency and small worldness. No significant three-way interactions were observed among the factors for either global index. A significant two-way interaction was detected between consciousness state and GS condition for small worldness (pd = 99.96%). Subsequent Bayesian ANOVA revealed Bayes factors of $BF_{10}$(state) = 0.298 and $BF_{10}$(GS scenario) = 1.381e+16 for GS condition, indicating that the GS condition substantially influenced small worldness measures.

## Supplementary figures

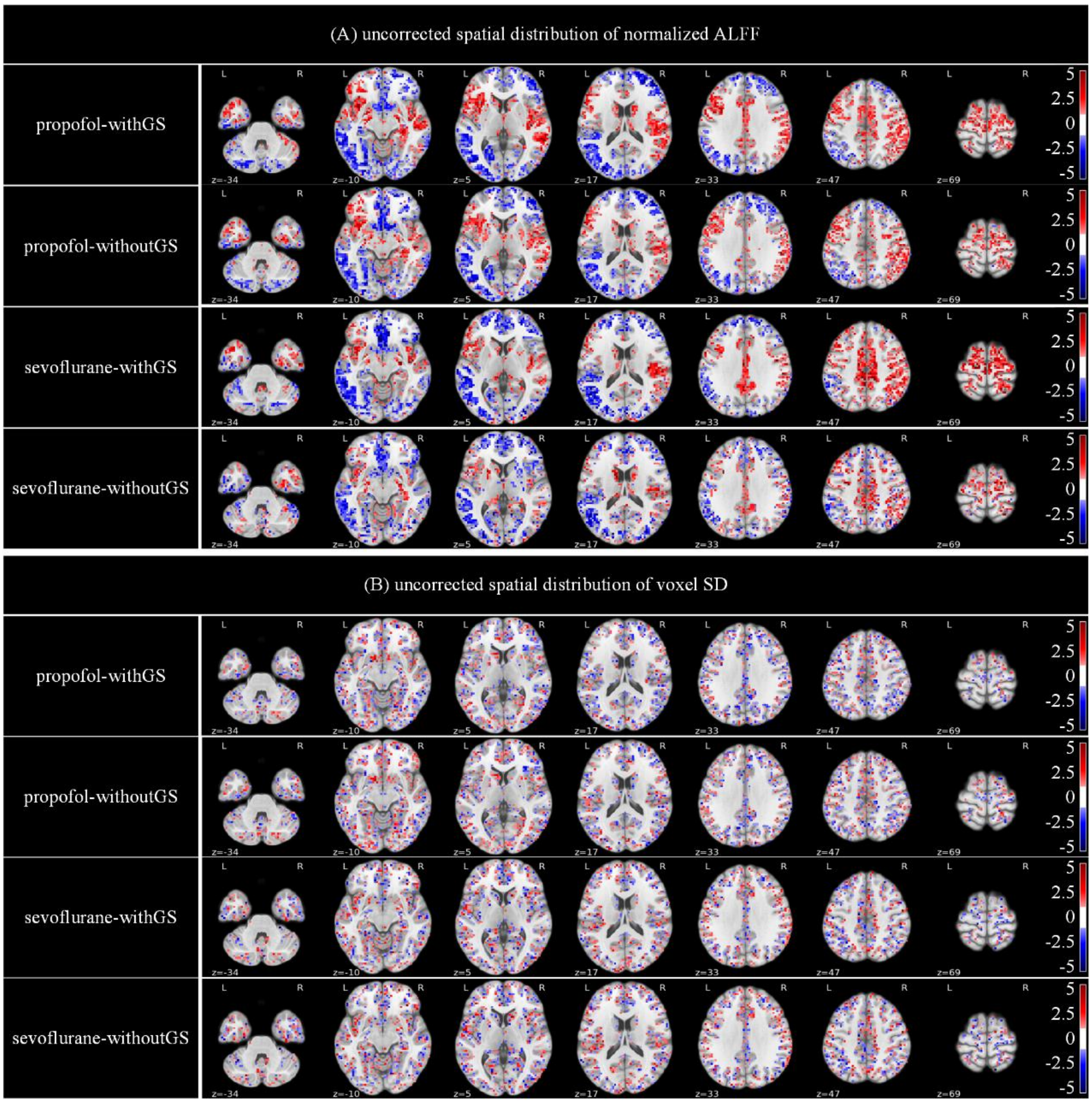

Figure S1. The voxel spatial distribution of normalized ALFF and voxel SD from wakefulness to unconscious. All the voxels were statistical using paired t-test (uncorrected). The color bar shows voxel-wise t-values (from 1.5 to 6) where red is for a decrease and blue is for an increase from baseline to unconscious state.

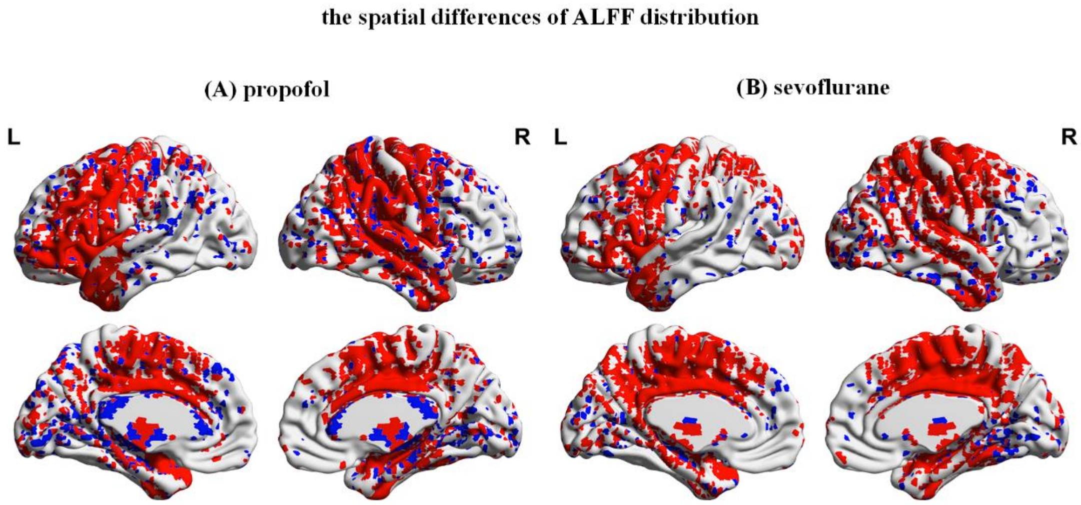


Figure S2. The spatial distributions difference of normalized ALFF between withGS and withoutGS for propofol and sevoflurane. The voxel in red represent the voxel show significance in withGS but not significance in withoutGS while voxel in blue is the opposite.

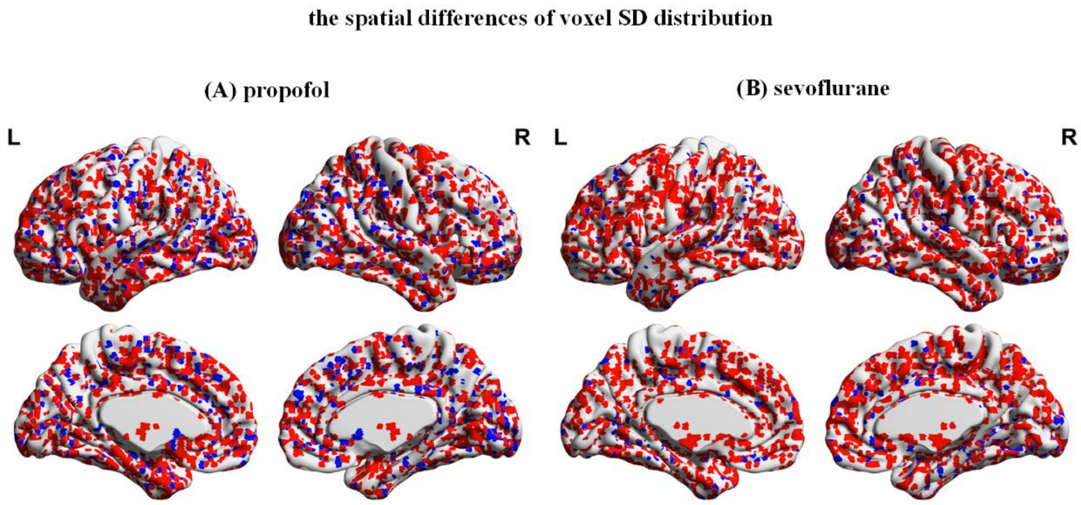

Figure S3. The spatial distributions difference of normalized SD between withGS and withoutGS for propofol and sevoflurane. The voxel in red represent the voxel show significance in withGS but not significance in withoutGS while voxel in blue is the opposite.

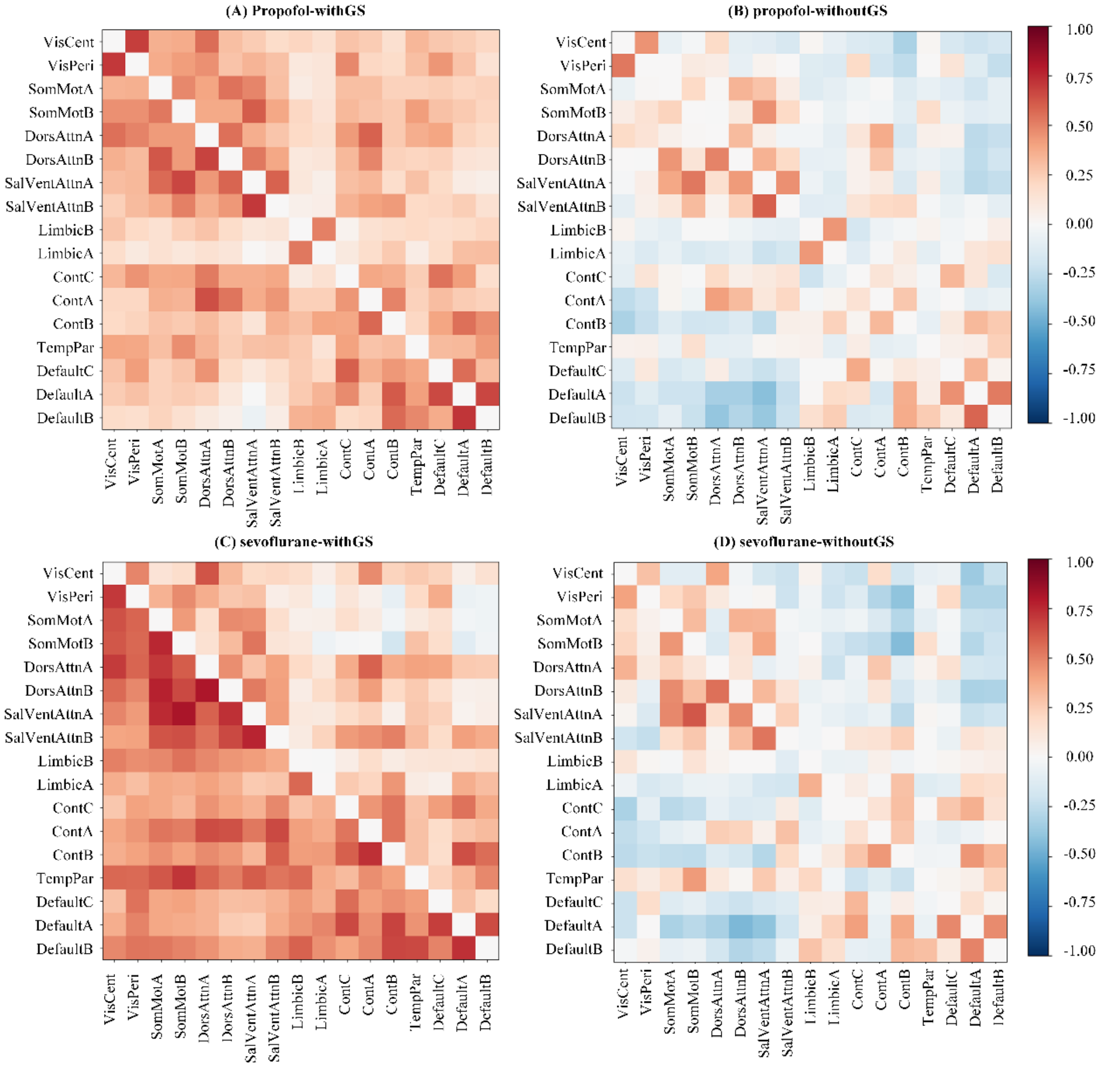


Figure S4. The FC matrix for propofol and sevoflurane in the scenario of withGS or withoutGS respectively. The lower triangle represents the FC matrix during the baseline, while the upper triangle represents the FC matrix during the unconscious state. For propofol and sevoflurane, both in baseline and unconscious state withGS, positive FC was found. While a lot of between network negative FC were found in withouGS.

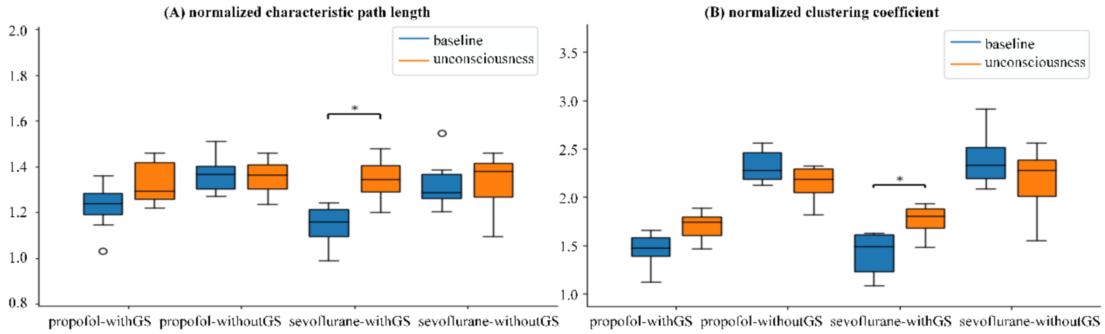


Figure S5. Group statistic boxplot of normalized characteristic path length and normalized clustering coefficient from baseline to unconscious state for propofol and sevoflurane respectively. The asterisks refer to significance level (*$BF_{10}$ > 3 between the states) for the Bayesian paired samples t tests.

## Supplementary tables

Table S1. Cost value for network indices calculation.

| | propofol | sevoflurane |
|---|---|---|
| baseline-withGS | 0.27 to 0.5 | 0.205 to 0.425 |
| unconsciousness-withGS | 0.245 to 0.39 | 0.22 to 0.405 |
| baseline-withoutGS | 0.095 to 0.465 | 0.075 to 0.485 |
| unconsciousness-withoutGS | 0.105 to 0.465 | 0.10 to 0.475 |

Table S2. $BF_{10}$, median, LowerCI and UpperCI for SD of GM average time series between baseline and unconscious state for propofol and sevoflurane.

| | drugs | GS scenario | BF10 | median | LowerCI | UpperCI |
|---|---|---|---|---|---|---|
| baseline-unconscious state | propofol | withGS | 3.892 | 0.0365 | 1.64E-02 | 0.0554 |
| baseline-unconscious state | propofol | withoutGS | 7.432 | 0.0135 | -6.31E-03 | 0.0331 |
| baseline-unconscious state | sevoflurane | withGS | 7.409 | 0.0657 | 4.42E-25 | 0.0859 |
| baseline-unconscious state | sevoflurane | withoutGS | 11.55 | 0.0206 | 1.07E-06 | 0.042 |

Table S3. $BF_{10}$, median, LowerCI and UpperCI for SD of GM average time series between withGS and withoutGS for propofol and sevoflurane.

| | drugs | state | BF10 | median | LowerCI | UpperCI |
|---|---|---|---|---|---|---|
| withGS-withoutGS | propofol | baseline | 0.509 | 0.00151 | 8.01E-02 | 0.0829 |
| withGS-withoutGS | propofol | unconscious state | 0.489 | 0.03434 | -0.0489 | 1.13E-01 |
| withGS-withoutGS | sevoflurane | baseline | 0.516 | 0.03967 | -5.19E-02 | 0.1252 |
| withGS-withoutGS | sevoflurane | unconscious state | 0.54 | 0.03497 | -5.56E-02 | 0.1204 |

Table S4. $BF_{10}$, median, LowerCI and UpperCI for voxel SD between baseline and unconscious state for propofol and sevoflurane.

| | drugs | GS scenario | BF10 | median | LowerCI | UpperCI |
|---|---|---|---|---|---|---|
| baseline-unconscious state | propofol | withGS | 0.366 | -0.000339 | -2.50E-03 | 0.00188 |
| baseline-unconscious state | propofol | withoutGS | 0.372 | -0.000383 | -2.59E-03 | 0.00169 |
| baseline-unconscious state | sevoflurane | withGS | 0.374 | -0.000123 | -2.45E-03 | 0.00226 |
| baseline-unconscious state | sevoflurane | withoutGS | 0.374 | 0.00009 | -2.18E-03 | 0.00244 |

Table S5. $BF_{10}$, median, LowerCI and UpperCI for voxel SD between withGS and withoutGS for propofol and sevoflurane.

| | drugs | state | BF10 | median | LowerCI | UpperCI |
|---|---|---|---|---|---|---|
| withGS-withoutGS | propofol | baseline | 1.34E-04 | -1.93E-03 | 0.0024 | 1.34E-04 |
| withGS-withoutGS | propofol | unconscious state | 8.77E-05 | -0.00204 | 2.24E-03 | 8.77E-05 |
| withGS-withoutGS | sevoflurane | baseline | 9.72E-05 | -2.33E-03 | 0.00233 | 9.72E-05 |
| withGS-withoutGS | sevoflurane | unconscious state | -3.13E-04 | -2.03E-03 | 0.00267 | -3.13E-04 |

Table S6. $BF_{10}$, median, LowerCI and UpperCI for normalize ALFF between baseline and unconscious state for propofol and sevoflurane.

| | drugs | GS scenario | BF10 | median | LowerCI | UpperCI |
|---|---|---|---|---|---|---|
| baseline-unconscious state | propofol | withGS | 0.366 | -0.000339 | -2.50E-03 | 0.00188 |
| baseline-unconscious state | propofol | withoutGS | 0.372 | -0.000383 | -2.59E-03 | 0.00169 |

| | | | | | | |
|---|---|---|---|---|---|---|
| baseline-unconscious state | sevoflurane | withGS | 0.374 | -0.000123 | -2.45E-03 | 0.00226 |
| baseline-unconscious state | sevoflurane | withoutGS | 0.374 | 0.00009 | -2.18E-03 | 0.00244 |

Table S7. $BF_{10}$, median, LowerCI and UpperCI for normalize ALFF between withGS and withoutGS for propofol and sevoflurane.

| | drugs | state | BF10 | median | LowerCI | UpperCI |
|---|---|---|---|---|---|---|
| withGS-withoutGS | propofol | baseline | 0.509 | 0.00151 | 8.01E-02 | 0.0829 |
| withGS-withoutGS | propofol | unconscious state | 0.489 | 0.03434 | -0.0489 | 1.13E-01 |
| withGS-withoutGS | sevoflurane | baseline | 0.516 | 0.03967 | -5.19E-02 | 0.1252 |
| withGS-withoutGS | sevoflurane | unconscious state | 0.54 | 0.03497 | -5.56E-02 | 0.1204 |

Table S8. $BF_{10}$, median, LowerCI and UpperCI for network average path length between baseline and unconscious state for propofol.

| | GS scenario | network | $BF_{10}$ | median | LowerCI | UpperCI |
|---|---|---|---|---|---|---|
| baseline-unconscious state | withGS | VisCent | 3.151 | -2.4 | -4.12E+00 | -0.74 |
| baseline-unconscious state | withGS | VisPeri | 2.105 | -2.68 | -4.39 | -0.978 |
| baseline-unconscious state | withGS | SomMotA | 2.989 | -2.62 | -4.39 | -1.014 |
| baseline-unconscious state | withGS | SomMotB | 2.556 | -3.14 | -4.81 | -1.47 |
| baseline-unconscious state | withGS | DorsAttnA | 5.371 | -2.5 | -4.26 | -0.875 |
| baseline-unconscious state | withGS | DorsAttnB | 3.967 | -3.21 | -4.91 | -1.574 |
| baseline-unconscious state | withGS | SalVentAttnA | 2.707 | -3.33 | -4.93 | -1.621 |
| baseline-unconscious state | withGS | SalVentAttnB | 2.285 | -3.47 | -5.15 | -1.763 |
| baseline-unconscious state | withGS | LimbicB | 8.52 | -4.21 | -5.88 | -2.511 |
| baseline-unconscious state | withGS | LimbicA | 2.461 | -2.95 | -4.60E+00 | -1.243 |
| baseline-unconscious state | withGS | ContC | 3.506 | -3.27 | -4.95 | -1.537 |
| baseline-unconscious state | withGS | ContA | 2.296 | -3.21 | -4.87 | -1.538 |
| baseline-unconscious state | withGS | ContB | 2.66 | -3.07 | -4.76 | -1.395 |
| baseline-unconscious state | withGS | TempPar | 2.828 | -2.52 | -4.21 | -0.817 |
| baseline-unconscious state | withGS | DefaultC | 5.004 | -3.29 | -4.9 | -1.587 |

| | | | | | | |
|---|---|---|---|---|---|---|
| baseline-unconscious state | withGS | DefaultA | 3.097 | -2.69 | -4.36 | -0.921 |
| baseline-unconscious state | withGS | DefaultB | 2.392 | -2.63 | -4.36 | -1.013 |
| baseline-unconscious state | withoutGS | VisCent | 2.616 | -3.17 | -4.80E+00 | -1.429 |
| baseline-unconscious state | withoutGS | VisPeri | 1.62 | -2.88 | -4.54 | -1.178 |
| baseline-unconscious state | withoutGS | SomMotA | 10.296 | -3.38 | -5.06 | -1.612 |
| baseline-unconscious state | withoutGS | SomMotB | 5.446 | -3.73 | -5.39 | -2.045 |
| baseline-unconscious state | withoutGS | DorsAttnA | 2.398 | -3.15 | -4.84 | -1.437 |
| baseline-unconscious state | withoutGS | DorsAttnB | 6.971 | -4.08 | -5376 | -2.371 |
| baseline-unconscious state | withoutGS | SalVentAttnA | 6.15 | -4.86 | -6.51 | -3.175 |
| baseline-unconscious state | withoutGS | SalVentAttnB | 3.393 | -4.22 | -5.96 | -2.579 |
| baseline-unconscious state | withoutGS | LimbicB | 6.157 | -4.97 | -6.62 | -3.233 |
| baseline-unconscious state | withoutGS | LimbicA | 3.296 | -3.43 | -5.14E+00 | -1.733 |
| baseline-unconscious state | withoutGS | ContC | 3.074 | -3.9 | -5.6 | -2.24 |
| baseline-unconscious state | withoutGS | ContA | 4.248 | -4.17 | -5.87 | -2.542 |
| baseline-unconscious state | withoutGS | ContB | 2 | -3.25 | -4.9 | -1.541 |
| baseline-unconscious state | withoutGS | TempPar | 1.453 | -2.32 | -4.05 | -0.651 |
| baseline-unconscious state | withoutGS | DefaultC | 2.656 | -3.78 | -5.52 | -2.118 |
| baseline-unconscious state | withoutGS | DefaultA | 2.248 | -2.83 | -4.49 | -1.098 |
| baseline-unconscious state | withoutGS | DefaultB | 2.458 | -3.05 | -4.77 | -1.39 |

Table S9. $BF_{10}$, median, LowerCI and UpperCI for network average path length between withGS and withoutGS for propofol.

| | GS scenario | network | $BF_{10}$ | median | LowerCI | UpperCI |
|---|---|---|---|---|---|---|
| withGS-withoutGS | baseline | VisCent | 167.774 | -3.86 | -5.55 | -2.093 |
| withGS-withoutGS | baseline | VisPeri | 126.588 | -4.29 | -6 | -2.574 |
| withGS-withoutGS | baseline | SomMotA | 395.411 | -3.44 | -5.15 | -1.759 |
| withGS-withoutGS | baseline | SomMotB | 113.156 | -3.32 | -5.01 | -1.639 |
| withGS-withoutGS | baseline | DorsAttnA | 133.985 | -3.49 | -5.21 | -1.78 |
| withGS-withoutGS | baseline | DorsAttnB | 35.5 | -2.97 | -1.265 | -4.69 |

| | | | | | | |
|---|---|---|---|---|---|---|
| withGS-withoutGS | baseline | SalVentAttnA | 113.045 | -2.49 | -4.16 | -0.761 |
| withGS-withoutGS | baseline | SalVentAttnB | 127.352 | -2.51 | -4.2 | -0.894 |
| withGS-withoutGS | baseline | LimbicB | 30.081 | -3.33 | -5.04 | -1.586 |
| withGS-withoutGS | baseline | LimbicA | 202.301 | -3.11 | -4.78 | -1.468 |
| withGS-withoutGS | baseline | ContC | 252.357 | -3.24 | -4.92 | -1.513 |
| withGS-withoutGS | baseline | ContA | 31.728 | -2.81 | -4.43 | -1.111 |
| withGS-withoutGS | baseline | ContB | 134.328 | -2.95 | -4.64 | -1.214 |
| withGS-withoutGS | baseline | TempPar | 118.871 | -3.58 | -5.33 | -1.909 |
| withGS-withoutGS | baseline | DefaultC | 91.973 | -3.39 | -5.04 | -1.686 |
| withGS-withoutGS | baseline | DefaultA | 323.751 | -3.03 | -4.76 | -1.344 |
| withGS-withoutGS | baseline | DefaultB | 115.402 | -2.78 | -4.53 | -1.113 |
| withGS-withoutGS | unconscious state | VisCent | 731.463 | -4.68 | -6.42 | -2.98 |
| withGS-withoutGS | unconscious state | VisPeri | 2212.339 | -4.51 | -6.19 | -2.755 |
| withGS-withoutGS | unconscious state | SomMotA | 594.394 | -4.19 | -5.87 | -2.467 |
| withGS-withoutGS | unconscious state | SomMotB | 762.204 | -3.9 | -5.58 | -2.199 |
| withGS-withoutGS | unconscious state | DorsAttnA | 669.233 | -4.11 | -5.84 | -2.432 |
| withGS-withoutGS | unconscious state | DorsAttnB | 455.088 | -3.8 | -5.52 | -2.13 |
| withGS-withoutGS | unconscious state | SalVentAttnA | 368.904 | -3.96 | -5.65 | -2.28 |
| withGS-withoutGS | unconscious state | SalVentAttnB | 55.583 | -3.27 | -4.94 | -1.593 |
| withGS-withoutGS | unconscious state | LimbicB | 66.747 | -4.06 | -5.75 | -2.349 |
| withGS-withoutGS | unconscious state | LimbicA | 227.636 | -3.59 | -5.2 | -1.835 |
| withGS-withoutGS | unconscious state | ContC | 142.603 | -3.88 | -5.51 | -2.148 |
| withGS-withoutGS | unconscious state | ContA | 73.198 | -3.77 | -5.47 | -2.06 |
| withGS-withoutGS | unconscious state | ContB | 201.462 | -3.11 | -4.76 | -1.434 |
| withGS-withoutGS | unconscious state | TempPar | 273.807 | -3.39 | -5.1 | -1.676 |
| withGS-withoutGS | unconscious state | DefaultC | 215.12 | -3.88 | -5.63 | -2.211 |
| withGS-withoutGS | unconscious state | DefaultA | 151.472 | -3.21 | -4.85 | -1.486 |
| withGS-withoutGS | unconscious state | DefaultB | 101.474 | -3.2 | -4.82 | -1.476 |

Table S10. $BF_{10}$, median, LowerCI and UpperCI for network average path length

between baseline and unconscious state for sevoflurane.

| | GS scenario | network | $BF_{10}$ | median | LowerCI | UpperCI |
|---|---|---|---|---|---|---|
| baseline-unconscious state | withGS | VisCent | 8.601 | -3.36 | -6.73 | -0.262 |
| baseline-unconscious state | withGS | VisPeri | 26.862 | -4.12 | -7.35 | -0.701 |
| baseline-unconscious state | withGS | SomMotA | 15.734 | -4.31 | -7.54 | -1.089 |
| baseline-unconscious state | withGS | SomMotB | 8.592 | -4.41 | -7.69 | -1.069 |
| baseline-unconscious state | withGS | DorsAttnA | 5.394 | -3 | -6.36 | 0.227 |
| baseline-unconscious state | withGS | DorsAttnB | 5.735 | -4.2 | -7.49 | -0.852 |
| baseline-unconscious state | withGS | SalVentAttnA | 8.178 | -4.1 | -7.31 | -0.827 |
| baseline-unconscious state | withGS | SalVentAttnB | 6.396 | -4.17 | -7.38 | -0.878 |
| baseline-unconscious state | withGS | LimbicB | 10.307 | -5.85 | -9 | -2.558 |
| baseline-unconscious state | withGS | LimbicA | 2.703 | -3.88 | -7.11 | -0.554 |
| baseline-unconscious state | withGS | ContC | 4.02 | -3.84 | -7.08 | -0.366 |
| baseline-unconscious state | withGS | ContA | 3.946 | -4.41 | -7.63 | -0.993 |
| baseline-unconscious state | withGS | ContB | 3.881 | -3.83 | -7.2 | -0.605 |
| baseline-unconscious state | withGS | TempPar | 6.64 | -2.98 | -6.27 | 0.266 |
| baseline-unconscious state | withGS | DefaultC | 3.772 | -3.89 | -7.36 | -0.724 |
| baseline-unconscious state | withGS | DefaultA | 5.727 | -3.58 | -6.76 | -0.166 |
| baseline-unconscious state | withGS | DefaultB | 6.24 | -3.77 | -7.16 | -0.629 |
| baseline-unconscious state | withoutGS | VisCent | 1.229 | -5.05 | -8.24 | -1.804 |
| baseline-unconscious state | withoutGS | VisPeri | 1.364 | -5.67 | -9.15 | -2.564 |
| baseline-unconscious state | withoutGS | SomMotA | 1.465 | -6.33 | -9.52 | -3.029 |
| baseline-unconscious state | withoutGS | SomMotB | 2.15 | -6.02 | -9.13 | -2.618 |
| baseline-unconscious state | withoutGS | DorsAttnA | 1.167 | -5.24 | -8.31 | -1.826 |
| baseline-unconscious state | withoutGS | DorsAttnB | 1.782 | -6.66 | -9.86 | -3.259 |
| baseline-unconscious state | withoutGS | SalVentAttnA | 1.969 | -6.57 | -9.69 | -3.239 |
| baseline-unconscious state | withoutGS | SalVentAttnB | 2.26 | -6.44 | -9.71 | -3.129 |
| baseline-unconscious state | withoutGS | LimbicB | 1.65 | -7.86 | -11.17 | -4.615 |
| baseline-unconscious state | withoutGS | LimbicA | 1.325 | -5.72 | -9.03 | -2.533 |

| | | | | | | |
|---|---|---|---|---|---|---|
| baseline-unconscious state | withoutGS | ContC | 1.737 | -6.63 | -9.9 | -3.299 |
| baseline-unconscious state | withoutGS | ContA | 1.79 | -5.72 | -8.87 | -2.413 |
| baseline-unconscious state | withoutGS | ContB | 1.513 | -6.02 | -9.16 | -2.599 |
| baseline-unconscious state | withoutGS | TempPar | 1.363 | -4.27 | -7.45 | -0.9 |
| baseline-unconscious state | withoutGS | DefaultC | 1.635 | -6.13 | -9.37 | -2.866 |
| baseline-unconscious state | withoutGS | DefaultA | 1.254 | -5.71 | -8.86 | -2.295 |
| baseline-unconscious state | withoutGS | DefaultB | 1.301 | -5.44 | -8.75 | -2.175 |

Table S11. $BF_{10}$, median, LowerCI and UpperCI for network average path length between withGS and withoutGS for sevoflurane.

| | GS scenario | network | BF10 | median | LowerCI | UpperCI |
|---|---|---|---|---|---|---|
| withGS-withoutGS | baseline | VisCent | 143.876 | -4.4 | -7.74E+00 | -1.1647 |
| withGS-withoutGS | baseline | VisPeri | 241.207 | -4.4 | -7.68 | -1.1675 |
| withGS-withoutGS | baseline | SomMotA | 68.742 | -3.83 | -7.11 | -0.54 |
| withGS-withoutGS | baseline | SomMotB | 359.936 | -3.51 | -6.78 | -0.335 |
| withGS-withoutGS | baseline | DorsAttnA | 1233.569 | -3.66 | -6.97 | -0.4082 |
| withGS-withoutGS | baseline | DorsAttnB | 32.619 | -3.49 | -6.67 | -0.135 |
| withGS-withoutGS | baseline | SalVentAttnA | 63.801 | -3.13 | -6.44 | 0.1156 |
| withGS-withoutGS | baseline | SalVentAttnB | 27.72 | -3.24 | -6.44 | 0.0509 |
| withGS-withoutGS | baseline | LimbicB | 22.642 | -3.76 | -7.05 | -0.4216 |
| withGS-withoutGS | baseline | LimbicA | 64.461 | -3.4 | -6.64E+00 | -0.0468 |
| withGS-withoutGS | baseline | ContC | 22.184 | -3 | -6.31 | 0.2952 |
| withGS-withoutGS | baseline | ContA | 75.55 | -3.78 | -7.1 | -0.5965 |
| withGS-withoutGS | baseline | ContB | 329.845 | -2.91 | -6.18 | 0.4081 |
| withGS-withoutGS | baseline | TempPar | 212.001 | -3.65 | -6.97 | -0.5122 |
| withGS-withoutGS | baseline | DefaultC | 73.087 | -3.34 | -6.51 | -0.0833 |
| withGS-withoutGS | baseline | DefaultA | 206.197 | -3.13 | -6.44 | 0.0985 |
| withGS-withoutGS | baseline | DefaultB | 1375.419 | -3.18 | -6.58 | -0.0492 |
| withGS-withoutGS | unconscious state | VisCent | 2.578 | -5.81 | -9.08 | -2.49E+00 |

| | | | | | | |
|---|---|---|---|---|---|---|
| withGS-withoutGS | unconscious state | VisPeri | 2.231 | -5.99 | -9.18 | -2.6416 |
| withGS-withoutGS | unconscious state | SomMotA | 1.761 | -5.87 | -9.18 | -2.5574 |
| withGS-withoutGS | unconscious state | SomMotB | 1.648 | -5.12 | -8.5 | -2.0034 |
| withGS-withoutGS | unconscious state | DorsAttnA | 2.532 | -5.87 | -9.12 | -2.6465 |
| withGS-withoutGS | unconscious state | DorsAttnB | 2.856 | -5.83 | -8.96 | -2.4218 |
| withGS-withoutGS | unconscious state | SalVentAttnA | 2.125 | -5.61 | -8.75 | -2.2393 |
| withGS-withoutGS | unconscious state | SalVentAttnB | 3.165 | -5.49 | -8.62 | -2.0053 |
| withGS-withoutGS | unconscious state | LimbicB | 1.686 | -5.79 | -8.99 | -2.3997 |
| withGS-withoutGS | unconscious state | LimbicA | 2.969 | -5.25 | -8.42 | -1.97E+00 |
| withGS-withoutGS | unconscious state | ContC | 2.888 | -5.79 | -9.03 | -2.54 |
| withGS-withoutGS | unconscious state | ContA | 4.048 | -5.14 | -8.36 | -1.8562 |
| withGS-withoutGS | unconscious state | ContB | 2.698 | -5.13 | -8.42 | -1.8341 |
| withGS-withoutGS | unconscious state | TempPar | 2.672 | -4.89 | -8.3 | -1.7628 |
| withGS-withoutGS | unconscious state | DefaultC | 2.43 | -5.62 | -8.98 | -2.4599 |
| withGS-withoutGS | unconscious state | DefaultA | 1.915 | -5.23 | -8.39 | -1.9219 |
| withGS-withoutGS | unconscious state | DefaultB | 1.862 | -4.84 | -8.04 | -1.6607 |

Table S12. $BF_{10}$, median, LowerCI and UpperCI for network average clustering coefficient between baseline and unconscious state for propofol.

| | GS scenario | network | BF10 | median | LowerCI | UpperCI |
|---|---|---|---|---|---|---|
| baseline-unconscious state | withGS | VisCent | 1.368 | 0.0575 | 1.46E-02 | 0.0993 |
| baseline-unconscious state | withGS | VisPeri | 1.093 | 0.0618 | 0.019476 | 0.1044 |
| baseline-unconscious state | withGS | SomMotA | 1.086 | 0.0512 | 0.009634 | 0.0947 |
| baseline-unconscious state | withGS | SomMotB | 1.27 | 0.0767 | 0.033406 | 0.1197 |
| baseline-unconscious state | withGS | DorsAttnA | 1.705 | 0.0705 | 0.027559 | 0.1133 |
| baseline-unconscious state | withGS | DorsAttnB | 2.922 | 0.0657 | 0.024309 | 0.1084 |
| baseline-unconscious state | withGS | SalVentAttnA | 2.64 | 0.01031 | 0.060545 | 0.1459 |
| baseline-unconscious state | withGS | SalVentAttnB | 2.334 | 0.0808 | 0.037984 | 0.1227 |
| baseline-unconscious state | withGS | LimbicB | 0.987 | 0.0715 | 0.028655 | 0.1141 |

| | | | | | | |
|---|---|---|---|---|---|---|
| baseline-unconscious state | withGS | LimbicA | 1.311 | 0.0654 | 2.37E-02 | 0.108 |
| baseline-unconscious state | withGS | ContC | 1.796 | 0.0868 | 0.043644 | 0.1287 |
| baseline-unconscious state | withGS | ContA | 4.673 | 0.0716 | 0.029632 | 0.1134 |
| baseline-unconscious state | withGS | ContB | 1.373 | 0.0635 | 0.020891 | 0.1056 |
| baseline-unconscious state | withGS | TempPar | 1.447 | 0.0615 | 0.020068 | 0.1055 |
| baseline-unconscious state | withGS | DefaultC | 6.908 | 0.0816 | 0.039098 | 0.1246 |
| baseline-unconscious state | withGS | DefaultA | 3.009 | 0.081 | 0.040386 | 0.1241 |
| baseline-unconscious state | withGS | DefaultB | 1.814 | 0.0677 | 0.026599 | 0.1109 |
| baseline-unconscious state | withoutGS | VisCent | 0.641 | 0.0305 | -1.12E-02 | 0.0748 |
| baseline-unconscious state | withoutGS | VisPeri | 0.445 | 0.0188 | -0.024212 | 0.0617 |
| baseline-unconscious state | withoutGS | SomMotA | 1.988 | 0.0386 | -0.02062 | 0.0819 |
| baseline-unconscious state | withoutGS | SomMotB | 2.452 | 0.0559 | 0.013864 | 0.0987 |
| baseline-unconscious state | withoutGS | DorsAttnA | 1.479 | 0.0338 | -0.009949 | 0.0755 |
| baseline-unconscious state | withoutGS | DorsAttnB | 2.541 | 0.0472 | 0.005321 | 0.0902 |
| baseline-unconscious state | withoutGS | SalVentAttnA | 5.966 | 0.0706 | 0.026937 | 0.1126 |
| baseline-unconscious state | withoutGS | SalVentAttnB | 3.635 | 0.0474 | 0.00377 | 0.0889 |
| baseline-unconscious state | withoutGS | LimbicB | 3.538 | 0.0417 | -0.000648 | 0.0845 |
| baseline-unconscious state | withoutGS | LimbicA | 3.372 | 0.0375 | -5.24E-03 | 0.0796 |
| baseline-unconscious state | withoutGS | ContC | 3.767 | 0.0437 | 0.001792 | 0.0858 |
| baseline-unconscious state | withoutGS | ContA | 3.09 | 0.0403 | -0.001138 | 0.0835 |
| baseline-unconscious state | withoutGS | ContB | 0.777 | 0.0249 | -0.018909 | 0.067 |
| baseline-unconscious state | withoutGS | TempPar | 0.587 | 0.0321 | -0.010034 | 0.0755 |
| baseline-unconscious state | withoutGS | DefaultC | 4.047 | 0.0503 | 0.008363 | 0.0928 |
| baseline-unconscious state | withoutGS | DefaultA | 1.986 | 0.0553 | 0.11939 | 0.0972 |
| baseline-unconscious state | withoutGS | DefaultB | 0.82 | 0.037 | -0.005154 | 0.0789 |

Table S13. $BF_{10}$, median, LowerCI and UpperCI for network average clustering coefficient between withGS and withoutGS for propofol.

| | GS scenario | network | BF10 | median | LowerCI | UpperCI |
|---|---|---|---|---|---|---|

| | | | | | | |
|---|---|---|---|---|---|---|
| withGS-withoutGS | baseline | VisCent | 3.51 | 0.089 | 4.57E-02 | 0.1317 |
| withGS-withoutGS | baseline | VisPeri | 2.552 | 0.088 | 0.045364 | 0.1301 |
| withGS-withoutGS | baseline | SomMotA | 5.7 | 0.1025 | 0.060695 | 0.1458 |
| withGS-withoutGS | baseline | SomMotB | 5.846 | 0.0815 | 0.039354 | 0.1251 |
| withGS-withoutGS | baseline | DorsAttnA | 61.97 | 0.0918 | 0.04933 | 0.1341 |
| withGS-withoutGS | baseline | DorsAttnB | 33.6 | 0.0849 | 0.042068 | 0.1275 |
| withGS-withoutGS | baseline | SalVentAttnA | 10.89 | 0.0895 | 0.042847 | 0.1312 |
| withGS-withoutGS | baseline | SalVentAttnB | 9.058 | 0.0768 | 0.032879 | 0.1177 |
| withGS-withoutGS | baseline | LimbicB | 7.045 | 0.083 | 0.040867 | 0.1262 |
| withGS-withoutGS | baseline | LimbicA | 10.87 | 0.0914 | 0.049921 | 0.1345 |
| withGS-withoutGS | baseline | ContC | 3.826 | 0.0758 | 0.034735 | 0.119 |
| withGS-withoutGS | baseline | ContA | 21.56 | 0.0847 | 0.040398 | 0.1252 |
| withGS-withoutGS | baseline | ContB | 5.533 | 0.1012 | 0.057748 | 0.1428 |
| withGS-withoutGS | baseline | TempPar | 8.519 | 0.0829 | 0.040697 | 0.1258 |
| withGS-withoutGS | baseline | DefaultC | 12.75 | 0.0934 | 0.050427 | 0.1353 |
| withGS-withoutGS | baseline | DefaultA | 7.086 | 0.0777 | 0.034341 | 0.1206 |
| withGS-withoutGS | baseline | DefaultB | 8.391 | 0.088 | 0.045555 | 0.1309 |
| withGS-withoutGS | unconscious state | VisCent | 19.99 | 0.0616 | 0.01925 | 1.07E-01 |
| withGS-withoutGS | unconscious state | VisPeri | 13.47 | 0.0605 | 0.017503 | 0.1022 |
| withGS-withoutGS | unconscious state | SomMotA | 12.1 | 0.0593 | 0.017376 | 0.1017 |
| withGS-withoutGS | unconscious state | SomMotB | 11.26 | 0.05 | 0.009117 | 0.094 |
| withGS-withoutGS | unconscious state | DorsAttnA | 109.6 | 0.0535 | 0.010729 | 0.097 |
| withGS-withoutGS | unconscious state | DorsAttnB | 22.83 | 0.0552 | 0.012324 | 0.0963 |
| withGS-withoutGS | unconscious state | SalVentAttnA | 16.82 | 0.0587 | 0.015682 | 0.1006 |
| withGS-withoutGS | unconscious state | SalVentAttnB | 17.42 | 0.0507 | 0.007495 | 0.0925 |
| withGS-withoutGS | unconscious state | LimbicB | 28.34 | 0.0521 | 0.01135 | 0.0964 |
| withGS-withoutGS | unconscious state | LimbicA | 4.761 | 0.0495 | 0.006445 | 0.0909 |
| withGS-withoutGS | unconscious state | ContC | 30.42 | 0.0634 | 0.020627 | 0.1062 |
| withGS-withoutGS | unconscious state | ContA | 103.1 | 0.0635 | 0.021714 | 0.1067 |

| | | | | | | |
|---|---|---|---|---|---|---|
| withGS-withoutGS | unconscious state | ContB | 28.81 | 0.0641 | 0.022009 | 0.108 |
| withGS-withoutGS | unconscious state | TempPar | 29.58 | 0.064 | 0.021429 | 0.107 |
| withGS-withoutGS | unconscious state | DefaultC | 68.97 | 0.0609 | 0.018371 | 0.1036 |
| withGS-withoutGS | unconscious state | DefaultA | 15.73 | 0.0439 | 0.000187 | 0.085 |
| withGS-withoutGS | unconscious state | DefaultB | 20.25 | 0.0582 | 0.016077 | 0.1019 |

Table S14. $BF_{10}$, median, LowerCI and UpperCI for network average clustering coefficient between baseline and unconscious state for sevoflurane.

| | GS scenario | network | BF10 | median | LowerCI | UpperCI |
|---|---|---|---|---|---|---|
| baseline-unconscious state | withGS | VisCent | 3.296 | 0.113 | 6.35E-02 | 0.1583 |
| baseline-unconscious state | withGS | VisPeri | 19.232 | 0.1614 | 0.11587 | 0.2108 |
| baseline-unconscious state | withGS | SomMotA | 2.826 | 0.0801 | 0.03192 | 0.1273 |
| baseline-unconscious state | withGS | SomMotB | 5.856 | 0.1324 | 0.08328 | 0.1788 |
| baseline-unconscious state | withGS | DorsAttnA | 10.881 | 0.1295 | 0.07634 | 0.1748 |
| baseline-unconscious state | withGS | DorsAttnB | 15.061 | 0.1092 | 0.06171 | 0.1587 |
| baseline-unconscious state | withGS | SalVentAttnA | 4.195 | 0.1273 | 0.08136 | 0.1759 |
| baseline-unconscious state | withGS | SalVentAttnB | 3.343 | 0.1022 | 0.05262 | 0.1487 |
| baseline-unconscious state | withGS | LimbicB | 1.974 | 0.1178 | 0.06688 | 0.1634 |
| baseline-unconscious state | withGS | LimbicA | 4.622 | 0.1263 | 7.56E-02 | 0.1724 |
| baseline-unconscious state | withGS | ContC | 16.562 | 0.1406 | 0.09586 | 0.1918 |
| baseline-unconscious state | withGS | ContA | 17.359 | 0.1072 | 0.05824 | 0.1542 |
| baseline-unconscious state | withGS | ContB | 4.696 | 0.1103 | 0.06151 | 0.1565 |
| baseline-unconscious state | withGS | TempPar | 13.022 | 0.1222 | 0.07319 | 0.1681 |
| baseline-unconscious state | withGS | DefaultC | 12.428 | 0.1578 | 0.10932 | 0.2048 |
| baseline-unconscious state | withGS | DefaultA | 3.345 | 0.0983 | 0.05028 | 0.1453 |
| baseline-unconscious state | withGS | DefaultB | 2.182 | 0.0998 | 0.05254 | 0.1485 |
| baseline-unconscious state | withoutGS | VisCent | 1.576 | 0.0648 | 1.50E-02 | 0.11 |
| baseline-unconscious state | withoutGS | VisPeri | 3.899 | 0.0785 | 0.03192 | 0.1273 |
| baseline-unconscious state | withoutGS | SomMotA | 1.187 | 0.0524 | 0.00542 | 0.1 |

| | | | | | | |
|---|---|---|---|---|---|---|
| baseline-unconscious state | withoutGS | SomMotB | 1.834 | 0.0579 | 0.01046 | 0.1073 |
| baseline-unconscious state | withoutGS | DorsAttnA | 2.555 | 0.0537 | 0.00804 | 0.1014 |
| baseline-unconscious state | withoutGS | DorsAttnB | 3.065 | 0.0531 | 0.00547 | 0.1006 |
| baseline-unconscious state | withoutGS | SalVentAttnA | 1.633 | 0.0605 | 0.01529 | 0.1098 |
| baseline-unconscious state | withoutGS | SalVentAttnB | 1.452 | 0.0406 | -0.00577 | 0.0894 |
| baseline-unconscious state | withoutGS | LimbicB | 0.732 | 0.0354 | -0.01299 | 0.0804 |
| baseline-unconscious state | withoutGS | LimbicA | 0.468 | 0.0243 | -2.31E-01 | 0.0728 |
| baseline-unconscious state | withoutGS | ContC | 4.428 | 0.0577 | 0.01026 | 0.1062 |
| baseline-unconscious state | withoutGS | ContA | 4.994 | 0.0425 | -0.00472 | 0.0896 |
| baseline-unconscious state | withoutGS | ContB | 0.971 | 0.0405 | -0.00795 | 0.0871 |
| baseline-unconscious state | withoutGS | TempPar | 1.317 | 0.0311 | -0.0158 | 0.0779 |
| baseline-unconscious state | withoutGS | DefaultC | 3.762 | 0.0636 | 0.01668 | 0.1118 |
| baseline-unconscious state | withoutGS | DefaultA | 0.505 | 0.0268 | -0.0208 | 0.0743 |
| baseline-unconscious state | withoutGS | DefaultB | 0.455 | 0.0205 | -0.02578 | 0.0702 |

Table S15. $BF_{10}$, median, LowerCI and UpperCI for network average clustering coefficient between withGS and withoutGS for sevoflurane.

| | GS scenario | network | BF10 | median | LowerCI | UpperCI |
|---|---|---|---|---|---|---|
| withGS-withoutGS | baseline | VisCent | 34.945 | 0.1241 | 7.68E-02 | 0.1739 |
| withGS-withoutGS | baseline | VisPeri | 22.784 | 0.1453 | 0.098368 | 0.1934 |
| withGS-withoutGS | baseline | SomMotA | 38.818 | 0.1057 | 0.058668 | 0.0154 |
| withGS-withoutGS | baseline | SomMotB | 13.82 | 0.1345 | 0.084214 | 0.1788 |
| withGS-withoutGS | baseline | DorsAttnA | 21.751 | 0.1451 | 0.097034 | 0.1922 |
| withGS-withoutGS | baseline | DorsAttnB | 65.709 | 0.1243 | 0.076984 | 0.1704 |
| withGS-withoutGS | baseline | SalVentAttnA | 33.022 | 0.1381 | 0.090867 | 0.1868 |
| withGS-withoutGS | baseline | SalVentAttnB | 56.72 | 0.1168 | 0.067822 | 0.1631 |
| withGS-withoutGS | baseline | LimbicB | 14.167 | 0.1463 | 0.099326 | 0.1937 |
| withGS-withoutGS | baseline | LimbicA | 16.551 | 0.1453 | 9.14E-02 | 0.1938 |
| withGS-withoutGS | baseline | ContC | 43.843 | 0.1319 | 0.085572 | 0.182 |

| | | | | | | |
|---|---|---|---|---|---|---|
| withGS-withoutGS | baseline | ContA | 515.059 | 0.1178 | 0.070574 | 0.1656 |
| withGS-withoutGS | baseline | ContB | 473.522 | 0.1229 | 0.075014 | 0.1706 |
| withGS-withoutGS | baseline | TempPar | 58.678 | 0.1409 | 0.094015 | 0.1887 |
| withGS-withoutGS | baseline | DefaultC | 58.904 | 0.1599 | 0.114164 | 0.2085 |
| withGS-withoutGS | baseline | DefaultA | 98.781 | 0.1239 | 0.076469 | 0.1704 |
| withGS-withoutGS | baseline | DefaultB | 164.986 | 0.1349 | 0.088126 | 0.1835 |
| withGS-withoutGS | unconscious state | VisCent | 14.338 | 0.0761 | 0.028851 | 1.24E-01 |
| withGS-withoutGS | unconscious state | VisPeri | 4.71 | 0.0622 | 0.015912 | 0.1103 |
| withGS-withoutGS | unconscious state | SomMotA | 12.103 | 0.0782 | 0.029774 | 0.1251 |
| withGS-withoutGS | unconscious state | SomMotB | 5.225 | 0.0602 | 0.011271 | 0.1067 |
| withGS-withoutGS | unconscious state | DorsAttnA | 19.109 | 0.0692 | 0.022416 | 0.118 |
| withGS-withoutGS | unconscious state | DorsAttnB | 30.233 | 0.0671 | 0.019797 | 0.1145 |
| withGS-withoutGS | unconscious state | SalVentAttnA | 16.841 | 0.0716 | 0.02434 | 0.1199 |
| withGS-withoutGS | unconscious state | SalVentAttnB | 79.875 | 0.0563 | 0.006819 | 0.1017 |
| withGS-withoutGS | unconscious state | LimbicB | 22.357 | 0.0639 | 0.016149 | 0.111 |
| withGS-withoutGS | unconscious state | LimbicA | 25.291 | 0.043 | -5.44E-03 | 0.0907 |
| withGS-withoutGS | unconscious state | ContC | 16.272 | 0.0491 | -0.000338 | 0.0958 |
| withGS-withoutGS | unconscious state | ContA | 61.133 | 0.0538 | 0.007325 | 0.1018 |
| withGS-withoutGS | unconscious state | ContB | 42.692 | 0.0539 | 0.007876 | 0.1024 |
| withGS-withoutGS | unconscious state | TempPar | 20.371 | 0.0494 | 0.003251 | 0.0966 |
| withGS-withoutGS | unconscious state | DefaultC | 13.329 | 0.066 | 0.01658 | 0.1112 |
| withGS-withoutGS | unconscious state | DefaultA | 61.018 | 0.0518 | 0.003106 | 0.0987 |
| withGS-withoutGS | unconscious state | DefaultB | 64.539 | 0.0556 | 0.008034 | 0.1021 |

Table S16. $BF_{10}$, median, LowerCI and UpperCI for network average local efficiency between baseline and unconscious state for propofol.

| | GS scenario | network | BF10 | median | LowerCI | UpperCI |
|---|---|---|---|---|---|---|
| baseline-unconscious state | withGS | VisCent | 1.418 | 0.0726 | 2.57E-02 | 0.1181 |
| baseline-unconscious state | withGS | VisPeri | 1.337 | 0.0752 | 0.02778 | 0.1222 |

| | | | | | | |
|---|---|---|---|---|---|---|
| baseline-unconscious state | withGS | SomMotA | 1.199 | 0.0526 | 0.00453 | 0.0975 |
| baseline-unconscious state | withGS | SomMotB | 1.318 | 0.0872 | 0.03996 | 0.134 |
| baseline-unconscious state | withGS | DorsAttnA | 1.874 | 0.0841 | 0.03712 | 0.1296 |
| baseline-unconscious state | withGS | DorsAttnB | 3.821 | 0.0816 | 0.03388 | 0.1283 |
| baseline-unconscious state | withGS | SalVentAttnA | 3.03 | 0.1161 | 0.06807 | 0.1624 |
| baseline-unconscious state | withGS | SalVentAttnB | 2.71 | 0.0898 | 0.04045 | 0.1342 |
| baseline-unconscious state | withGS | LimbicB | 1.582 | 0.0993 | 0.05299 | 0.1458 |
| baseline-unconscious state | withGS | LimbicA | 1.78 | 0.0878 | 4.31E-02 | 0.1353 |
| baseline-unconscious state | withGS | ContC | 2.32 | 0.0992 | 0.05225 | 0.1461 |
| baseline-unconscious state | withGS | ContA | 6.868 | 0.0917 | 0.04507 | 0.1379 |
| baseline-unconscious state | withGS | ContB | 1.945 | 0.0857 | 0.03831 | 0.1299 |
| baseline-unconscious state | withGS | TempPar | 1.83 | 0.086 | 0.03882 | 0.1332 |
| baseline-unconscious state | withGS | DefaultC | 5.437 | 0.0937 | 0.04727 | 0.1409 |
| baseline-unconscious state | withGS | DefaultA | 2.942 | 0.1011 | 0.05436 | 0.1473 |
| baseline-unconscious state | withGS | DefaultB | 2.255 | 0.0915 | 0.04517 | 0.1381 |
| baseline-unconscious state | withoutGS | VisCent | 1.038 | 0.0473 | -1.34E-03 | 0.0913 |
| baseline-unconscious state | withoutGS | VisPeri | 0.625 | 0.0341 | -0.01221 | 0.0812 |
| baseline-unconscious state | withoutGS | SomMotA | 4.903 | 0.0791 | 0.03231 | 0.1276 |
| baseline-unconscious state | withoutGS | SomMotB | 3.574 | 0.0667 | 0.02074 | 0.1136 |
| baseline-unconscious state | withoutGS | DorsAttnA | 1.947 | 0.0491 | 0.00293 | 0.0971 |
| baseline-unconscious state | withoutGS | DorsAttnB | 4.586 | 0.0063 | 0.001912 | 0.1127 |
| baseline-unconscious state | withoutGS | SalVentAttnA | 9.719 | 0.0946 | 0.04748 | 0.1422 |
| baseline-unconscious state | withoutGS | SalVentAttnB | 4.817 | 0.0616 | 0.01613 | 0.109 |
| baseline-unconscious state | withoutGS | LimbicB | 8.42 | 0.0679 | 0.02045 | 0.1154 |
| baseline-unconscious state | withoutGS | LimbicA | 17.452 | 0.0621 | 1.69E-02 | 0.1098 |
| baseline-unconscious state | withoutGS | ContC | 4.04 | 0.0585 | 0.01001 | 0.1041 |
| baseline-unconscious state | withoutGS | ContA | 6.173 | 0.063 | 0.01513 | 0.1076 |
| baseline-unconscious state | withoutGS | ContB | 2.096 | 0.0494 | 0.00254 | 0.0965 |
| baseline-unconscious state | withoutGS | TempPar | 0.873 | 0.042 | -0.00382 | 0.0885 |

| | | | | | | |
|---|---|---|---|---|---|---|
| baseline-unconscious state | withoutGS | DefaultC | 4.022 | 0.0683 | 0.02326 | 0.1156 |
| baseline-unconscious state | withoutGS | DefaultA | 3.421 | 0.0752 | 0.02705 | 0.1223 |
| baseline-unconscious state | withoutGS | DefaultB | 2.034 | 0.0636 | 0.0169 | 0.1103 |

Table S17. $BF_{10}$, median, LowerCI and UpperCI for network average local efficiency between withGS and withoutGS for propofol.

| | GS scenario | network | BF10 | median | LowerCI | UpperCI |
|---|---|---|---|---|---|---|
| withGS-withoutGS | baseline | VisCent | 3.466 | 0.1 | 4.97E-02 | 0.1434 |
| withGS-withoutGS | baseline | VisPeri | 3.388 | 0.1043 | 0.0576 | 0.1519 |
| withGS-withoutGS | baseline | SomMotA | 5.79 | 0.1062 | 0.06008 | 0.1521 |
| withGS-withoutGS | baseline | SomMotB | 6.574 | 0.0949 | 0.04886 | 0.1434 |
| withGS-withoutGS | baseline | DorsAttnA | 8.655 | 0.1149 | 0.0687 | 0.1635 |
| withGS-withoutGS | baseline | DorsAttnB | 35.645 | 0.0843 | 0.0365 | 0.1317 |
| withGS-withoutGS | baseline | SalVentAttnA | 8.886 | 0.0938 | 0.0463 | 0.1406 |
| withGS-withoutGS | baseline | SalVentAttnB | 11.816 | 0.0801 | 0.03281 | 0.1269 |
| withGS-withoutGS | baseline | LimbicB | 11.841 | 0.1026 | 0.05622 | 0.1501 |
| withGS-withoutGS | baseline | LimbicA | 14.209 | 0.098 | 5.08E-02 | 0.1448 |
| withGS-withoutGS | baseline | ContC | 7.06 | 0.1113 | 0.06481 | 0.158 |
| withGS-withoutGS | baseline | ContA | 22.087 | 0.0849 | 0.03875 | 0.1327 |
| withGS-withoutGS | baseline | ContB | 7.558 | 0.1004 | 0.05308 | 0.1469 |
| withGS-withoutGS | baseline | TempPar | 11.067 | 0.1148 | 0.06794 | 0.1615 |
| withGS-withoutGS | baseline | DefaultC | 21.317 | 0.0949 | 0.04817 | 0.1423 |
| withGS-withoutGS | baseline | DefaultA | 7.55 | 0.0921 | 0.04479 | 0.1387 |
| withGS-withoutGS | baseline | DefaultB | 7.269 | 0.0931 | 0.04473 | 0.1384 |
| withGS-withoutGS | unconscious state | VisCent | 31.808 | 0.0735 | 0.02632 | 1.19E-01 |
| withGS-withoutGS | unconscious state | VisPeri | 39.616 | 0.0643 | 0.0172 | 0.1117 |
| withGS-withoutGS | unconscious state | SomMotA | 26.009 | 0.0796 | 0.03238 | 0.1248 |
| withGS-withoutGS | unconscious state | SomMotB | 19.548 | 0.0745 | 0.02776 | 0.1204 |
| withGS-withoutGS | unconscious state | DorsAttnA | 98.149 | 0.0796 | 0.03466 | 0.1274 |

| | | | | | | |
|---|---|---|---|---|---|---|
| withGS-withoutGS | unconscious state | DorsAttnB | 35.31 | 0.0686 | 0.02372 | 0.1176 |
| withGS-withoutGS | unconscious state | SalVentAttnA | 29.894 | 0.0727 | 0.02556 | 0.1193 |
| withGS-withoutGS | unconscious state | SalVentAttnB | 33.49 | 0.0518 | 0.00381 | 0.0975 |
| withGS-withoutGS | unconscious state | LimbicB | 47.127 | 0.0707 | 0.02592 | 0.1192 |
| withGS-withoutGS | unconscious state | LimbicA | 11.413 | 0.0724 | 2.78E-02 | 0.1194 |
| withGS-withoutGS | unconscious state | ContC | 60.029 | 0.0708 | 0.02514 | 0.118 |
| withGS-withoutGS | unconscious state | ContA | 201.711 | 0.0553 | 0.00808 | 0.1024 |
| withGS-withoutGS | unconscious state | ContB | 25.128 | 0.0634 | 0.01703 | 0.1116 |
| withGS-withoutGS | unconscious state | TempPar | 24.008 | 0.0702 | 0.02297 | 0.1155 |
| withGS-withoutGS | unconscious state | DefaultC | 51.983 | 0.0695 | 0.02479 | 0.1183 |
| withGS-withoutGS | unconscious state | DefaultA | 22.04 | 0.00662 | 0.01965 | 0.1132 |
| withGS-withoutGS | unconscious state | DefaultB | 22.316 | 0.0652 | 0.01804 | 0.1125 |

Table S18. $BF_{10}$, median, LowerCI and UpperCI for network average local efficiency between baseline and unconscious state for sevoflurane.

| | GS scenario | network | BF10 | median | LowerCI | UpperCI |
|---|---|---|---|---|---|---|
| baseline-unconscious state | withGS | VisCent | 7.106 | 0.1595 | 1.07E-01 | 0.211 |
| baseline-unconscious state | withGS | VisPeri | 51.354 | 0.1951 | 0.14319 | 0.245 |
| baseline-unconscious state | withGS | SomMotA | 12.722 | 0.17 | 0.11903 | 0.223 |
| baseline-unconscious state | withGS | SomMotB | 7.497 | 0.169 | 0.11789 | 0.222 |
| baseline-unconscious state | withGS | DorsAttnA | 13.575 | 0.1635 | 0.11337 | 0.216 |
| baseline-unconscious state | withGS | DorsAttnB | 16.977 | 0.1342 | 0.08031 | 0.184 |
| baseline-unconscious state | withGS | SalVentAttnA | 7.407 | 0.167 | 0.11289 | 0.217 |
| baseline-unconscious state | withGS | SalVentAttnB | 4.008 | 0.1287 | 0.07692 | 0.181 |
| baseline-unconscious state | withGS | LimbicB | 9.903 | 0.1868 | 0.13399 | 0.238 |
| baseline-unconscious state | withGS | LimbicA | 6.878 | 0.1561 | 1.06E-01 | 0.21 |
| baseline-unconscious state | withGS | ContC | 20.15 | 0.0918 | 0.03812 | 0.143 |
| baseline-unconscious state | withGS | ContA | 33.29 | 0.0704 | 0.01941 | 0.124 |
| baseline-unconscious state | withGS | ContB | 7.264 | 0.1576 | 0.10836 | 0.212 |

| | | | | | | |
|---|---|---|---|---|---|---|
| baseline-unconscious state | withGS | TempPar | 12.447 | 0.1637 | 0.11201 | 0.216 |
| baseline-unconscious state | withGS | DefaultC | 12.522 | 0.1772 | 0.12585 | 0.23 |
| baseline-unconscious state | withGS | DefaultA | 7.236 | 0.1577 | 0.10701 | 0.209 |
| baseline-unconscious state | withGS | DefaultB | 5.626 | 0.1556 | 0.10446 | 0.208 |
| baseline-unconscious state | withoutGS | VisCent | 2.41 | 0.0836 | 3.37E-02 | 0.136 |
| baseline-unconscious state | withoutGS | VisPeri | 7.609 | 0.0989 | 0.04746 | 0.15 |
| baseline-unconscious state | withoutGS | SomMotA | 2.642 | 0.0832 | 0.03189 | 0.136 |
| baseline-unconscious state | withoutGS | SomMotB | 3.467 | 0.0793 | 0.02746 | 0.13 |
| baseline-unconscious state | withoutGS | DorsAttnA | 3.189 | 0.0712 | 0.02092 | 0.123 |
| baseline-unconscious state | withoutGS | DorsAttnB | 5.273 | 0.0784 | 0.02682 | 0.131 |
| baseline-unconscious state | withoutGS | SalVentAttnA | 3.348 | 0.0929 | 0.03817 | 0.143 |
| baseline-unconscious state | withoutGS | SalVentAttnB | 2.386 | 0.0648 | 0.01156 | 0.116 |
| baseline-unconscious state | withoutGS | LimbicB | 2.057 | 0.0711 | 0.01867 | 0.122 |
| baseline-unconscious state | withoutGS | LimbicA | 0.972 | 0.0569 | 3.74E-03 | 0.108 |
| baseline-unconscious state | withoutGS | ContC | 7.323 | 0.1785 | 0.12813 | 0.231 |
| baseline-unconscious state | withoutGS | ContA | 6.957 | 0.1439 | 0.09149 | 0.196 |
| baseline-unconscious state | withoutGS | ContB | 2.039 | 0.071 | 0.0177 | 0.124 |
| baseline-unconscious state | withoutGS | TempPar | 2.129 | 0.0561 | 0.00607 | 0.11 |
| baseline-unconscious state | withoutGS | DefaultC | 4.944 | 0.0855 | 0.03365 | 0.137 |
| baseline-unconscious state | withoutGS | DefaultA | 1.113 | 0.0658 | 0.01512 | 0.118 |
| baseline-unconscious state | withoutGS | DefaultB | 0.979 | 0.0569 | 0.00481 | 0.109 |

Table S19. $BF_{10}$, median, LowerCI and UpperCI for network average local efficiency between withGS and withoutGS for sevoflurane.

| | GS scenario | network | BF10 | median | LowerCI | UpperCI |
|---|---|---|---|---|---|---|
| withGS-withoutGS | baseline | VisCent | 42.867 | 0.1582 | 1.05E-01 | 0.21 |
| withGS-withoutGS | baseline | VisPeri | 37.635 | 0.1701 | 0.11768 | 0.22 |
| withGS-withoutGS | baseline | SomMotA | 40.837 | 0.1718 | 0.11776 | 0.222 |
| withGS-withoutGS | baseline | SomMotB | 17.068 | 0.1563 | 0.10544 | 0.208 |

| | | | | | | |
|---|---|---|---|---|---|---|
| withGS-withoutGS | baseline | DorsAttnA | 36.02 | 0.1805 | 0.232 | 0.12804 |
| withGS-withoutGS | baseline | DorsAttnB | 134.849 | 0.129 | 0.07872 | 0.182 |
| withGS-withoutGS | baseline | SalVentAttnA | 41.767 | 0.1536 | 0.10091 | 0.205 |
| withGS-withoutGS | baseline | SalVentAttnB | 60.742 | 0.1287 | 0.07688 | 0.18 |
| withGS-withoutGS | baseline | LimbicB | 31.606 | 0.1793 | 0.12797 | 0.231 |
| withGS-withoutGS | baseline | LimbicA | 46.334 | 0.1627 | 1.10E-01 | 0.214 |
| withGS-withoutGS | baseline | ContC | 45.431 | 0.1532 | 0.10208 | 0.204 |
| withGS-withoutGS | baseline | ContA | 481.208 | 0.1349 | 0.187 | 0.08255 |
| withGS-withoutGS | baseline | ContB | 111.572 | 0.1507 | 0.10033 | 0.204 |
| withGS-withoutGS | baseline | TempPar | 89.259 | 0.0727 | 0.02089 | 0.125 |
| withGS-withoutGS | baseline | DefaultC | 62.34 | 0.1714 | 0.11789 | 0.221 |
| withGS-withoutGS | baseline | DefaultA | 83.688 | 0.1594 | 0.10883 | 0.213 |
| withGS-withoutGS | baseline | DefaultB | 108.555 | 0.1653 | 0.11144 | 0.215 |
| withGS-withoutGS | unconscious state | VisCent | 31.924 | 0.0836 | 0.03386 | 1.37E-01 |
| withGS-withoutGS | unconscious state | VisPeri | 10.359 | 0.0744 | 0.02231 | 0.125 |
| withGS-withoutGS | unconscious state | SomMotA | 21.248 | 0.0837 | 0.03313 | 0.137 |
| withGS-withoutGS | unconscious state | SomMotB | 7.042 | 0.0666 | 0.01414 | 0.118 |
| withGS-withoutGS | unconscious state | DorsAttnA | 44.303 | 0.0888 | 0.03725 | 0.141 |
| withGS-withoutGS | unconscious state | DorsAttnB | 129.632 | 0.0732 | 0.02022 | 0.124 |
| withGS-withoutGS | unconscious state | SalVentAttnA | 24.447 | 0.0803 | 0.02891 | 0.133 |
| withGS-withoutGS | unconscious state | SalVentAttnB | 94.673 | 0.0651 | 0.0131 | 0.115 |
| withGS-withoutGS | unconscious state | LimbicB | 35.323 | 0.0649 | 0.01148 | 0.115 |
| withGS-withoutGS | unconscious state | LimbicA | 59.865 | 0.0636 | 9.68E-03 | 0.115 |
| withGS-withoutGS | unconscious state | ContC | 20.864 | 0.0659 | 0.0136 | 0.118 |
| withGS-withoutGS | unconscious state | ContA | 798.348 | 0.0607 | 0.00963 | 0.113 |
| withGS-withoutGS | unconscious state | ContB | 66.752 | 0.0635 | 0.01107 | 0.0635 |
| withGS-withoutGS | unconscious state | TempPar | 59.088 | 0.1809 | 0.12725 | 0.231 |
| withGS-withoutGS | unconscious state | DefaultC | 26.454 | 0.08 | 0.132 | 0.02902 |
| withGS-withoutGS | unconscious state | DefaultA | 76.95 | 0.0668 | 0.119 | 0.01659 |

| | | | | | | |
|---|---|---|---|---|---|---|
| withGS-withoutGS | unconscious state | DefaultB | 107.164 | 0.0672 | 0.01585 | 0.0672 |

Table S20. $BF_{10}$, median, LowerCI and UpperCI for global efficiency between baseline and unconscious state for propofol and sevoflurane.

| | drugs | GS scenario | BF10 | median | LowerCI | UpperCI |
|---|---|---|---|---|---|---|
| baseline-unconscious state | propofol | withGS | 5.288 | 0.0728 | 4.22E-02 | 0.104 |
| baseline-unconscious state | propofol | withoutGS | 4.775 | 0.0391 | 8.44E-03 | 0.0701 |
| baseline-unconscious state | sevoflurane | withGS | 13.655 | 0.1031 | 6.98E-02 | 0.1371 |
| baseline-unconscious state | sevoflurane | withoutGS | 3.439 | 0.0481 | 1.41E-02 | 0.0814 |

Table S21. $BF_{10}$, median, LowerCI and UpperCI for global efficiency between withGS and withoutGS for propofol and sevoflurane.

| | drugs | state | BF10 | median | LowerCI | UpperCI |
|---|---|---|---|---|---|---|
| withGS-withoutGS | propofol | baseline | 31.504 | 0.0807 | 4.97E-02 | 0.1118 |
| withGS-withoutGS | propofol | unconscious state | 74.506 | 0.0464 | 0.0145 | 7.71E-02 |
| withGS-withoutGS | sevoflurane | baseline | 94.682 | 0.1027 | 6.97E-02 | 0.1374 |
| withGS-withoutGS | sevoflurane | unconscious state | 217.253 | 0.0485 | 1.46E-02 | 0.0816 |

Table S22. $BF_{10}$, median, LowerCI and UpperCI for small worldness between baseline and unconscious state for propofol and sevoflurane.

| | drugs | GS scenario | BF10 | median | LowerCI | UpperCI |
|---|---|---|---|---|---|---|
| baseline-unconscious state | propofol | withGS | 1.291 | -0.094 | -1.79E-01 | -0.00455 |
| baseline-unconscious state | propofol | withoutGS | 2.073 | 0.1177 | 2.93E-02 | 0.20739 |
| baseline-unconscious state | sevoflurane | withGS | 1.448 | -0.0786 | -1.75E-01 | 0.01322 |
| baseline-unconscious state | sevoflurane | withoutGS | 5.391 | 0.1669 | 6.97E-02 | 0.25836 |

Table S23. $BF_{10}$, median, LowerCI and UpperCI for small worldness between withGS and withoutGS for propofol and sevoflurane.

| | drugs | state | BF10 | median | LowerCI | UpperCI |
|---|---|---|---|---|---|---|

| | | | | | | |
|---|---|---|---|---|---|---|
| withGS-withoutGS | propofol | baseline | 125715.772 | -0.51 | -6.02E-01 | -0.427 |
| withGS-withoutGS | propofol | unconscious state | 885.628 | -0.3 | -0.386 | -2.11E-01 |
| withGS-withoutGS | sevoflurane | baseline | 104.615 | -0.556 | -6.55E-01 | -0.462 |
| withGS-withoutGS | sevoflurane | unconscious state | 94.263 | -0.31 | -4.04E-01 | -0.212 |

Table S24. $BF_{10}$, median, LowerCI and UpperCI for normalized characteristic path length between baseline and unconscious state for propofol and sevoflurane.

| | drugs | GS scenario | BF10 | median | LowerCI | UpperCI |
|---|---|---|---|---|---|---|
| baseline-unconscious state | propofol | withGS | 0.808 | 0.011848 | -1.00E-01 | 0.1227 |
| baseline-unconscious state | propofol | withoutGS | 0.359 | -0.000767 | -1.27E-01 | 0.11841 |
| baseline-unconscious state | sevoflurane | withGS | 3.886 | -0.106491 | -2.21E-01 | 0.00474 |
| baseline-unconscious state | sevoflurane | withoutGS | 0.373 | -0.202294 | -3.22E-01 | -0.08199 |

Table S25. $BF_{10}$, median, LowerCI and UpperCI for normalized characteristic path length between withGS and withoutGS for propofol and sevoflurane.

| | drugs | state | BF10 | median | LowerCI | UpperCI |
|---|---|---|---|---|---|---|
| withGS-withoutGS | propofol | baseline | 5.424 | -0.1386 | -2.49E-01 | -0.0227 |
| withGS-withoutGS | propofol | unconscious state | 0.444 | -0.0226 | -0.136 | 8.93E-02 |
| withGS-withoutGS | sevoflurane | baseline | 2.175 | -0.1876 | -3.07E-01 | -0.0658 |
| withGS-withoutGS | sevoflurane | unconscious state | 0.388 | 0.0157 | -1.05E-01 | 0.1359 |

Table S26. $BF_{10}$, median, LowerCI and UpperCI for normalized clustering coefficient between baseline and unconscious state for propofol and sevoflurane.

| | drugs | GS scenario | BF10 | median | LowerCI | UpperCI |
|---|---|---|---|---|---|---|
| baseline-unconscious state | propofol | withGS | 1.296 | -0.244 | -4.92E-01 | 0.00609 |
| baseline-unconscious state | propofol | withoutGS | 0.684 | 0.18 | -7.61E-02 | 0.41431 |
| baseline-unconscious state | sevoflurane | withGS | 3.56 | -0.346 | -6.01E-01 | -0.06128 |
| baseline-unconscious state | sevoflurane | withoutGS | 0.616 | 0.23 | -4.75E-02 | 0.48986 |

Table S27. $BF_{10}$, median, LowerCI and UpperCI for normalized clustering coefficient between withGS and withoutGS for propofol and sevoflurane.

| | drugs | state | BF10 | median | LowerCI | UpperCI |
|---|---|---|---|---|---|---|
| withGS-withoutGS | propofol | baseline | 2951.736 | -0.862 | -1.12E+00 | -0.62 |
| withGS-withoutGS | propofol | unconscious state | 2432.101 | -0.446 | -0.7 | -2.07E-01 |
| withGS-withoutGS | sevoflurane | baseline | 16.039 | -0.984 | -1.25E+00 | -0.718 |
| withGS-withoutGS | sevoflurane | unconscious state | 7.768 | -0.406 | -6.67E-01 | -0.131 |

## References

Abraham, Alexandre, Fabian Pedregosa, Michael Eickenberg, Philippe Gervais, Andreas Mueller, Jean Kossaifi, Alexandre Gramfort, Bertrand Thirion, and Gael Varoquaux. 2014. “Machine Learning for Neuroimaging with Scikit-Learn.” Frontiers in Neuroinformatics 8. https://doi.org/10.3389/fninf.2014.00014.

Avants, B. B., C. L. Epstein, M. Grossman, and J. C. Gee. 2008. “Symmetric Diffeomorphic Image Registration with Cross-Correlation: Evaluating Automated Labeling of Elderly and Neurodegenerative Brain.” Medical Image Analysis 12 (1): 26–41. https://doi.org/10.1016/j.media.2007.06.004.

Behzadi, Yashar, Khaled Restom, Joy Liau, and Thomas T. Liu. 2007. “A Component Based Noise Correction Method (CompCor) for BOLD and Perfusion Based fMRI.” NeuroImage 37 (1): 90–101. https://doi.org/10.1016/j.neuroimage.2007.04.042.

Cox, Robert W., and James S. Hyde. 1997. “Software Tools for Analysis and Visualization of fMRI Data.” NMR in Biomedicine 10 (4-5): 171–78. https://doi.org/10.1002/(SICI)1099-1492(199706/08)10:4/5<171::AID-NBM453>3.0.CO;2-L.

Dale, Anders M., Bruce Fischl, and Martin I. Sereno. 1999. “Cortical Surface-Based Analysis: I. Segmentation and Surface Reconstruction.” NeuroImage 9 (2): 179–94.

https://doi.org/10.1006/nimg.1998.0395.
Esteban, Oscar, Ross Blair, Christopher J. Markiewicz, Shoshana L. Berleant, Craig Moodie, Feilong Ma, Ayse Ilkay Isik, et al. 2018. "fMRIPrep." Software. https://doi.org/10.5281/zenodo.852659.
Esteban, Oscar, Christopher Markiewicz, Ross W Blair, Craig Moodie, Ayse Ilkay Isik, Asier Erramuzpe Aliaga, James Kent, et al. 2018. "fMRIPrep: A Robust Preprocessing Pipeline for Functional MRI." Nature Methods. https://doi.org/10.1038/s41592-018-0235-4.
Fonov, VS, AC Evans, RC McKinstry, CR Almli, and DL Collins. 2009. "Unbiased Nonlinear Average Age-Appropriate Brain Templates from Birth to Adulthood." NeuroImage 47, Supplement 1: S102. https://doi.org/10.1016/S1053-8119(09)70884-5.
Gorgolewski, K., C. D. Burns, C. Madison, D. Clark, Y. O. Halchenko, M. L. Waskom, and S. Ghosh. 2011. "Nipype: A Flexible, Lightweight and Extensible Neuroimaging Data Processing Framework in Python." Frontiers in Neuroinformatics 5: 13. https://doi.org/10.3389/fninf.2011.00013.
Gorgolewski, Krzysztof J., Oscar Esteban, Christopher J. Markiewicz, Erik Ziegler, David Gage Ellis, Michael Philipp Notter, Dorota Jarecka, et al. 2018. "Nipype." Software. https://doi.org/10.5281/zenodo.596855.
Greve, Douglas N, and Bruce Fischl. 2009. "Accurate and Robust Brain Image Alignment Using Boundary-Based Registration." NeuroImage 48 (1): 63–72. https://doi.org/10.1016/j.neuroimage.2009.06.060.
Jenkinson, Mark, Peter Bannister, Michael Brady, and Stephen Smith. 2002. "Improved Optimization for the Robust and Accurate Linear Registration and Motion Correction of Brain Images." NeuroImage 17 (2): 825–41. https://doi.org/10.1006/nimg.2002.1132.
Klein, Arno, Satrajit S. Ghosh, Forrest S. Bao, Joachim Giard, Yrjö Häme, Eliezer Stavsky, Noah Lee, et al. 2017. "Mindboggling Morphometry of Human Brains." PLOS Computational Biology 13 (2): e1005350. https://doi.org/10.1371/journal.pcbi.1005350.
Lanczos, C. 1964. "Evaluation of Noisy Data." Journal of the Society for Industrial and Applied Mathematics Series B Numerical Analysis 1 (1): 76–85.

https://doi.org/10.1137/0701007.
Power, Jonathan D., Anish Mitra, Timothy O. Laumann, Abraham Z. Snyder, Bradley L. Schlaggar, and Steven E. Petersen. 2014. “Methods to Detect, Characterize, and Remove Motion Artifact in Resting State fMRI.” NeuroImage 84 (Supplement C): 320–41. https://doi.org/10.1016/j.neuroimage.2013.08.048.
Satterthwaite, Theodore D., Mark A. Elliott, Raphael T. Gerraty, Kosha Ruparel, James Loughead, Monica E. Calkins, Simon B. Eickhoff, et al. 2013. “An improved framework for confound regression and filtering for control of motion artifact in the preprocessing of resting-state functional connectivity data.” NeuroImage 64 (1): 240–56. https://doi.org/10.1016/j.neuroimage.2012.08.052.
Tustison, N. J., B. B. Avants, P. A. Cook, Y. Zheng, A. Egan, P. A. Yushkevich, and J. C. Gee. 2010. “N4itk: Improved N3 Bias Correction.” IEEE Transactions on Medical Imaging 29 (6): 1310–20. https://doi.org/10.1109/TMI.2010.2046908.
Zhang, Y., M. Brady, and S. Smith. 2001. “Segmentation of Brain MR Images Through a Hidden Markov Random Field Model and the Expectation-Maximization Algorithm.” IEEE Transactions on Medical Imaging 20 (1): 45–57. https://doi.org/10.1109/42.906424.
